# Dynamic changes in the date palm fruit proteome during development and ripening

**DOI:** 10.1038/hortres.2014.39

**Published:** 2014-08-06

**Authors:** Claudius Marondedze, Christoph Gehring, Ludivine Thomas

**Affiliations:** 1 Biological and Environmental Sciences & Engineering Division, King Abdullah University of Science and Technology, Thuwal 23955-6900, Saudi Arabia; 2 Bioscience and Bioengineering Core Facility, King Abdullah University of Science and Technology, Thuwal 23955-6900, Saudi Arabia

## Abstract

Date palm (*Phoenix dactylifera*) is an economically important fruit tree in the Middle East and North Africa and is characterized by large cultivar diversity, making it a good model for studies on fruit development and other important traits. Here *in gel* comparative proteomics combined with tandem mass spectrometry were used to study date fruit development and ripening. Total proteins were extracted using a phenol-based protocol. A total of 189 protein spots were differentially regulated (*p*≤0.05). The identified proteins were classified into 14 functional categories. The categories with the most proteins were ‘disease and defense’ (16.5%) and ‘metabolism’ (15.4%). Twenty-nine proteins have not previously been identified in other fleshy fruits and 64 showed contrasting expression patterns in other fruits. Abundance of most proteins with a role in abiotic stress responses increased during ripening with the exception of heat shock proteins. Proteins with a role in anthocyanin biosynthesis, glycolysis, tricarboxylic acid cycle and cell wall degradation were upregulated particularly from the onset of ripening and during ripening. In contrast, expression of pentose phosphate- and photosynthesis-related proteins decreased during fruit maturation. Although date palm is considered a climacteric species, the analysis revealed downregulation of two enzymes involved in ethylene biosynthesis, suggesting an ethylene-independent ripening of ‘Barhi’ fruits. In summary, this proteomics study provides insights into physiological processes during date fruit development and ripening at the systems level and offers a reference proteome for the study of regulatory mechanisms that can inform molecular and biotechnological approaches to further improvements of horticultural traits including fruit quality and yield.

## Introduction

Date palm (*Phoenix dactylifera*) is a perennial, dioecious monocot that is highly heterozygous.^1^ Dates have been an agriculturally and economically important fruit crop for centuries in the Middle East and Northern Africa.^2^ They are not only a staple for millions of people,^3^ but they also have potential health benefits due to their high nutrient content and bioactive compounds including polyphenols such as flavonoids, tannins and phenolics.^4–6^ The date industry generated about 7.5 million tons worldwide in 2012 and Saudi Arabia, the third major producer after Egypt and Iran, grows over 400 cultivars.^7^ ‘Sukkary’ and ‘Barhi’ are the two most popular cultivars;^8,9^ however, their productivity is irregular. Therefore, breeding strategies, including the use of molecular tools, offer the potential to contribute towards improving productivity and quality.

Like other major commercial fruits such as apples (*Malus* × *domestica*) and bananas (*Musa acuminata*), dates are classified as climacteric fruits as the ripening processes are associated with a concomitant burst of ethylene and a peak in respiration.^10,11^ Fruit development and ripening consist of complex biological processes associated with major metabolic pathways and cellular processes undergoing gradual switches from cell division to energy, nutrient and metabolite storage during cell expansion and eventually starch degradation during ripening.^12–14^ Understanding date palm fruit development biology at the molecular level is still in its infancy, but the recent release of the date genome enables a more systematic investigation of fruit development.^9,^^15–17^ Proteomics in combination with phenotypical and physiological data offers a promising approach to further characterize fruit development and quality traits. Comparative proteomics has furthered our understanding of biological processes during fruit development, ripening, postharvest and senescence in apple,^18^ tomato (*Solanum lycopersicum*),^19,20^ strawberry (*Fragaria* × *ananassa*),^21^ grape (*Vitis vinifera*),^22–24^ peach (*Prunus persica*)^25,26^ and apricot (*Prunus armeniaca*).^27^ In date palm, comparative proteomics has been applied only to the analysis of somatic and zygotic embryos,^28^ but not to date fruit tissues. The objective of this study was to generate comparative proteomic profiles for inferring of date hypanthium protein changes during development and ripening with a view to provide new insights into date fruit biology and support efforts to improve date horticulture.

## Materials and methods

### Plant material

Date palm fruits (cultivar ‘Barhi’) were collected at Thuwal, Province of Makkah, on the shores of the Red Sea in Saudi Arabia at four developmental stages: 25 days after pollination (DAP; S1), 70 DAP (MD), 110 DAP (NTR) and 125 DAP (RIPE-Khalal stage). The optimal date of harvest, designated as S1, range from 1 to 25 April 2011 and 2012. This was based on fruit size, shape, color and the historical harvest date for this cultivar. Fruits were immediately rinsed with sterile distilled water, frozen in liquid nitrogen, transported to the laboratory and stored at −80 °C. For comparative proteomics, three biological repetitions comprising 10-pooled fruits harvested over two consecutive years for each stage were used.

### Protein extraction

Proteins were extracted from date hypanthia by phenol extraction as described previously,^29^ with some modifications. Fruit skin and innermost layers including the seed were removed prior to extraction. Approximately 5 g of frozen tissue was ground twice for 5 s each to a fine powder in 5 mL of cold extraction buffer (1% (w/v) polyvinylpolypyrrolidone, 0.7 M sucrose, 0.1 M KCl, 0.5 M Tris-HCl pH 7.5, 500 mM ethylenediaminetetraacetic acid, 1 mM phenylmethanesulfonylfluoride, 2% (v/v) β-mercaptoethanol) on ice using a PowerGen™ Model 125 homogenizer (Fisher Scientific, Waltham, MA, USA). Volume was adjusted to 15 mL and the mixture homogenized for 30 min at 4 °C. The mixture was homogenized for a further 30 min at 4 °C after addition of an equal volume of 0.5 M Tris-HCl pH 7.5-saturated phenol and then centrifuged at 10 000*g* for 30 min at 4 °C. The upper phenol phase was used to extract proteins twice in extraction buffer. The final phenol phase was precipitated with five volumes of saturated ammonium acetate in 100% (v/v) methanol overnight at −20 °C. Proteins were pelleted by centrifugation at 10 000*g* for 30 min at 4 °C and washed once in 100% (v/v) ice-cold methanol and subsequently three times in 100% (v/v) ice-cold acetone. Each wash was followed by centrifugation at 10 000*g* for 10 min at 4 °C to collect proteins, which were finally air-dried and solubilized in isoelectric focusing buffer (7 M urea, 2 M thiourea, 4% (w/v) 3-[(3-cholamidopropyl)dimethylammonio]-1-propanesulfonate, 1% (v/v) IPG buffer pH 3–10). Proteins were quantified by Bradford^30^ using Quick Start Bradford reagent (Bio-Rad, Hercules, CA, USA) and bovine serum albumin as standard. Quality was assessed by one-dimensional gel electrophoresis.

### Comparative two-dimensional gel electrophoresis (2-DE) and protein identification

Proteins (50 μg) were used to rehydrate a 7-cm-long linear IPG strip, pH range 4–7 (GE Healthcare, Pittsburgh, PA, USA). 2-DE, SYPRO^®^ Ruby (Molecular Probes, Eugene, OR, USA) staining and imaging were performed as detailed previously.^31^ Image analysis with Delta 2D v4.2 (DECODON, Greifswald, Germany) and trypsin digestion of differentially expressed spots were carried out as reported previously.^32^ Digested peptides were dried and stored at −20 °C. Peptides were analyzed with an LTQ-Orbitrap Velos (Thermo-Scientific, Bremen, Germany) coupled to nanoelectrospray ion source (Proxeon Biosystems, Odense, Denmark) for liquid chromatography-tandem mass spectrometry (LC-MS/MS) as described previously.^32^ Raw data files were converted to mgf using Proteome Discoverer v1.2 (Thermo-Scientific). All spectra were submitted to a local MASCOT (Matrix Science, London, UK) server and searched against *Phoenix dactylifera* dataset downloaded from http://qatar-weill.cornell.edu/research/datepalmGenome/download.html (version 3) using a precursor mass tolerance of ±10 ppm, a fragment ion mass tolerance of ±0.6 Da, and strict trypsin specificity allowing up to one missed cleavage, carbamidomethyl on cysteine residues as fixed modification, and oxidation of methionine residues and phosphorylation of serine, threonine and tyrosine residues as variable modifications. Proteins were considered positive if the Mascot score was over the 95% confidence limit (≥26 for plant). Data was validated with Scaffold v4 (Proteome Software, Portland, OR, USA). The dataset presented here only include proteins and peptides with ≥95% probability each and ≤1% false discovery rate. Scaffold PTM (Proteome Software) was used to identify and position phosphorylation and oxidation posttranslational modifications (PTMs).

### Bioinformatics analysis for functional enrichment and protein classification

Blast2GO (http://www.blast2go.org; v2.6.6)^33^ was used for gene ontology (GO) functional enrichment analysis. Blast-p search of identified protein sequences was performed against the NCBI non-redundant database with a minimum expectation value of 1×10^−3^. Annotations were made with default parameters: pre-eValue-Hit-Filter at 1×10^−6^, cut-off was set at 55 and GO weight at 5. Annotation was augmented using Annotation Expander (ANNEX) and the addition of GO terms associated with functional domains derived from scanning the InterPro database. Functional classification was performed as described previously.^34^

## Results and discussion

### Comparative 2-DE analysis and protein identification

**Figure 1:**
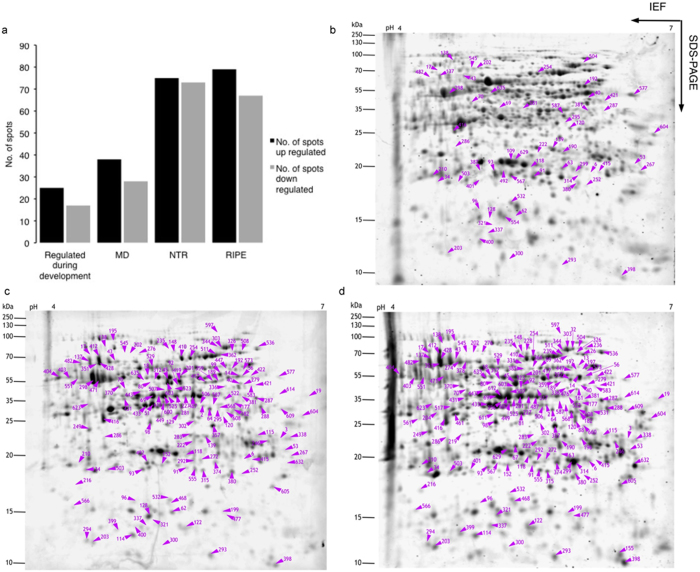
2-DE proteome maps of the date hypanthium showing differentially expressed spots. The relative molecular mass and p*I* are indicated on the left and at the top of the gels. Total soluble proteins (∼50 μg) from date hypanthium collected at different developmental stages were separated in the first dimension on immobilized linear pH 4–7 gradient and then on 12% acrylamide gels for the second dimension. Gels were stained with SYPRO^®^ Ruby and comparatively analyzed with Delta 2D (Decodon). **a** shows number of up- and downregulated spots overall and at each stage in comparison to S1. Differentially expressed spot numbers are shown at MD (**b**), NTR (**c**) and RIPE (**d**) in comparison to S1.

‘Barhi’ fruits, when harvested at RIPE (Khalal stage) before over-ripening (Rutab stage), are firm, yellow and contain about 30% moisture.^35^ A phenol-based extraction method yielded a protein fraction that produced high-quality 2-DE maps with minimal streaking in the acidic pH range and well-resolved protein spots (Figure 1). Reference gel images displayed distinct dissimilarities in terms of spot abundance and distribution among the four developmental stages (S1, MD, NTR and RIPE). Differences were observed particularly over p*I* range 4–5. The principal component analysis showed a closer correlation between biological replicates than between developmental stages (Supplementary File 1). The Delta 2D analysis revealed an average of 960 unique spots resolved on individual 2-DE and this is comparable to recently reported fruit proteome profiles.^27,^^36–41^

**Figure 2:**
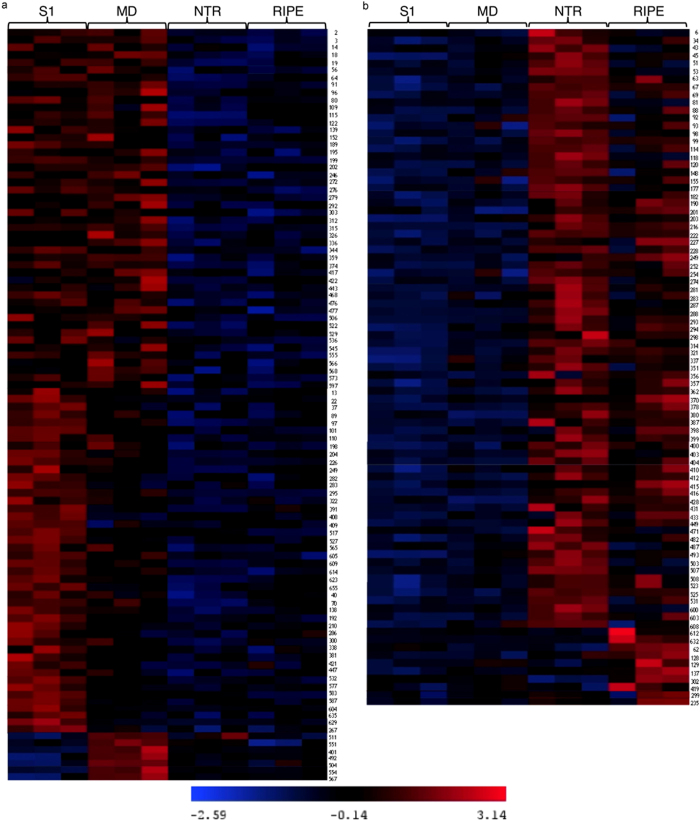
Heat maps of differentially expressed protein spots during fruit development and ripening. Clustered spots show different expression during fruit development according to the normalized spot pixel intensity in each replicate. **a** shows spots mainly downregulated from NTR and **b** shows spots mainly upregulated at NTR and RIPE.

The abundance of 189 protein spots was significantly altered (*p*≤0.01) with a minimum fold change of ±1.5 during the four developmental stages and these spots were selected for further analyses (Figure 1). Heat maps revealed major changes in protein expression over the course of date development and ripening. A first subset of spots was downregulated from NTR stage (Figure 2a), while a second subset was upregulated (Figure 2b). Of the 189 significantly altered spots, 171 were positively identified by LC-MS/MS corresponding to 193 unique proteins (Supplementary Files 2 and 3), while 18 spots (6, 51, 118, 139, 148, 152, 161, 204, 207, 219, 236, 276, 293, 315, 321, 399, 505 and 517) remained unidentified (Figure 1). Of the 171 identified spots, 68 (representing 82 proteins) and 78 (representing 96 proteins) were up- or downregulated at different times. A further 25 spots (representing 36 proteins) showed differential accumulation throughout development (Supplementary File 2).

### GO analysis of differentially expressed proteins

**Figure 3:**
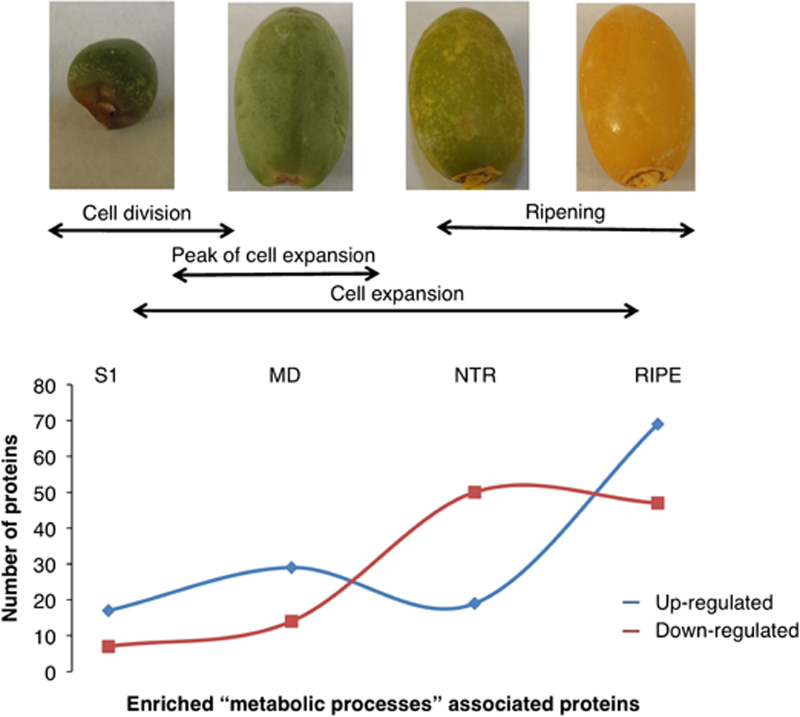
Analysis of enriched ‘metabolic processes’-associated proteins against each of the four developmental stages investigated.

GO analysis using Blast2GO facilitated classification of the 193 identified proteins. The GO analysis is a tool that allows inferring function based on enrichments in comparison to the total proteome. Among the 82 upregulated and 96 downregulated proteins during fruit development, the most enriched biological processes (*p*≤0.05) are ‘metabolic’ and ‘cellular processes’, ‘response to stimulus’ and ‘biological regulation’ (Supplementary File 4). The most enriched molecular functions (*p*≤0.05) included ‘catalytic activity’ and ‘binding’. Enrichments were also detected in cellular compartments ‘vesicle’, ‘fumarate reductase complex’ and ‘respiratory chain complex II’ (Supplementary File 4). GO terms detected were most markedly enriched at RIPE stage, suggesting occurrence of a distinct metabolic phase that could possibly serve as an indicator of developmental reprogramming (Figure 3).

The most represented GO term was ‘metabolic processes’ with 134 proteins differentially regulated during fruit development and ripening (Figure 3 and Supplementary File 4). In this category, abundance of 17 proteins consistently increased throughout development and ripening, while 29, 19 and 69 proteins were transiently upregulated only at MD, NTR and RIPE, respectively (Figure 3). In the ‘response to stress’ category, 22 proteins were upregulated throughout the entire development and ripening stages, while 29 proteins were transiently downregulated at NTR and RIPE (Supplementary File 4). The GO analysis of the date fruit-dependent proteome revealed that proteins from specific metabolic and cellular processes are induced at particular developmental stages suggesting maturation specific roles.

### Date-specific hypanthium proteins and their expression

Of the 193 identified proteins that were significantly differentially expressed (*p*≤0.01), we noted that 29 were specific to date fruit and, according to Bevan *et al*.,^34^ belong to 10 functional categories (Table 1). To date, these proteins have not been identified in any other fleshy fruits, possibly due to limited proteomic data available from fleshy fruits of monocot plants.^40,41^ However, identification of this set of proteins could also be due to the different stages at which fruits were collected and characterized. For example banana fruits were grouped into two stages, pre- and post-climacteric,^40^ while in the current study, four stages were considered, two before ripening (S1 and MD) and two after the onset of ripening (NTR and RIPE). An additional five proteins, proline iminopeptidase (spot 129), aspartyl-tRNA synthetase (spot 274), GDP dissociation inhibitor (spot 370), disproportionating enzyme (spot 385) and ornithine carbamoyltransferase (spot 566), which have been detected in other fleshy fruits, but not characterized as differentially expressed during development or ripening, were identified in this study (Table 1).

Sixty-four differentially expressed date proteins (Table 2) showed opposite transient accumulation to previously reported expression changes in fruits such as tomato,^19^ strawberry^21^ and apple.^14^ These proteins include 30s ribosomal protein s1 (spot 287), whose expression increased in date during development and ripening, particularly at NTR and RIPE, and aldo/keto reductase (spots 274, 378, 523 and 525) that was upregulated at NTR and RIPE. These proteins were reported to decrease in tomato,^19^ strawberry^21^ and apple^14^ during fruit development and ripening. Likewise, proteins involved in metabolism such as glucan endo-1,4-β-glucosidase (spot 192), energy metabolism-related proteins like transaldolase-like protein (spot 577) and transketolase 1 (spot 202), signal transduction proteins such as zinc finger (spot 587) and disease/defense protein, E3 ubiquitin ligase (spot 583) were downregulated at NTR and RIPE in the date hypanthium, but upregulated in papaya (*Carica papaya*),^42^ peach,^43^ mango (*Mangifera indica*)^44^ and grapes^45^ during fruit ripening. The different protein expression patterns noted here may indicate date specific development patterns and/or result from differing plant interactions with their respective environments.^46^

### Classification of development and ripening-specific proteins

**Figure 4:**
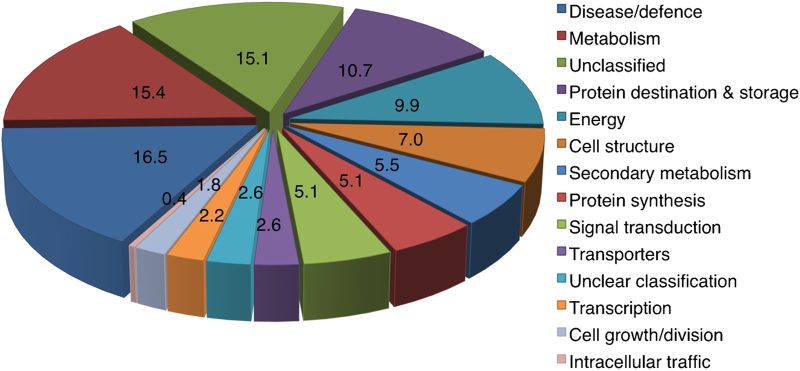
Classification of the differentially expressed proteins. Following 2-DE, protein spots were visualized by SYPRO^®^ Ruby staining and comparatively analyzed with Delta 2D (Decodon). Differentially expressed spots were identified using LC-MS/MS and classified into functional categories according to Ref. 34.

Based on UniProt (http://www.uniprot.org/), TAIR (http://www.arabidopsis.org/) and published literature, differentially expressed proteins were assigned to 14 categories previously described:^34^ ‘disease and defense’ (16.5%), ‘metabolism’ (15.4%), ‘unclassified’ (15.1%), ‘protein destination and storage’ (10.7%), ‘energy’ (9.9%), ‘cellular structure’ (7.0%), ‘secondary metabolism’ (5.5%), ‘signal transduction’ (5.1%), ‘protein synthesis’ (5.1%), ‘unclear classification’ (2.6%), ‘transporters’ (2.6%), ‘transcription’ (2.2%), ‘cell growth/division’ (1.8%) and ‘intracellular traffic’ (0.4%) (Figure 4 and Supplementary File 2). Some proteins have dual roles including ‘response to abiotic stress’ and this will be discussed in the sections below.

#### Response to abiotic stress

In this category, nine proteins were identified as date hypanthium-specific, 29 proteins showed contrasting accumulation patterns compared to other fruits and seven had similar patterns. Of the nine date hypanthium-specific proteins, expression of two transcription-related proteins, GATA transcription factor 25 (spot 302) and inducer of C-repeat-binding factor expression 1 DNA-binding transcription activator (spot 302) (Table 1), was reduced at NTR and later increased at RIPE in comparison to S1 stage. C-repeat-binding factor expression 1 is a transcription factor regulating expression of a number of genes with a role in abiotic stress responses including responses to cold,^47,48^ drought, high temperature and salt stress.^49,50^ The transient upregulation of C-repeat-binding factor expression 1 at NTR suggests that it might play a role in abiotic stress responses at the onset of date hypanthium ripening, a stage characterized by changes in metabolic activities and fruit physiology.

Two date-specific proteins involved in protein synthesis, group antigen polymerase (Gag-pol) polyprotein (spot 249) and aspartyl-tRNA synthetase (spot 274), were upregulated during ripening (Table 1). Aspartyl-tRNA synthetase, essential during the first step of translation, has been implicated in processes including response to cadmium ion and aspartyl-tRNA aminoacylation, protein maturation and response to salt stress. Its expression was also detected in apple and strawberry fruit extracts.^51^ In addition to Gag-pol polyprotein and aspartyl-tRNA synthetase, another protein identified in this category, elongation factor thermo unstable (EF-Tu; spots 431 and 433; Supplementary File 2) has been previously detected in maturing fruits. This protein belongs to the GTP binding EF-Tu family and plays an essential role in translational elongation. In plants, EF-Tu is involved in chaperone activity to protect protein aggregation in response to environmental stress, thereby facilitating degradation of N-terminally blocked proteins by the proteasome and triggering resistance to pathogenic bacteria.^52^ Over-expression of an EF-Tu gene has been shown to improve heat tolerance in maize (*Zea mays*).^53^ Similar to the upregulation observed in the current study, it was also detected as increasing during grape ripening.^24^

Further, five stress-responsive proteins classified in the category ‘protein destination and storage’ showed differential accumulation during development. Three proteins were detected as upregulated, proline iminopeptidase (spot 129) at NTR only, GDP dissociation inhibitor (spot 370) from the onset of ripening, and the ε-subunit chaperonin containing tailless complex protein (spot 531) at RIPE. Expression of two other proteins, proteasome subunit β type 7-A (spot 210) and nuclear transport (spot 300), decreased at all stages (Table 1). GDP dissociation inhibitor is involved in signaling on the plasma membrane and may be essential for actin reorganization.^54^ The protein was detected in young satsuma mandarins (*Citrus unshiu*)^55^ and its expression increased in peach fruits following post-harvest heat treatment.^43^ In the latter study, GDP dissociation inhibitor was proposed to play a vital role in maintenance of cell integrity during fruit ripening and senescence, and its upregulation at NTR and RIPE suggests a similar role in the date hypanthium.

Expression of aldose reductase (spot 401), a disease/defense-related protein and a member of the aldo-keto reductase family, increased at both MD and RIPE. Unlike other members of the family (e.g., aldo-keto reductase), this protein has not been reported as differentially expressed during development in other fruits. Aldose reductase catalyzes the Nicotinamide adenine dinucleotide phosphate-dependent reduction of carbonyl and aldehyde metabolites like the reduction of glucose to sorbitol.^56^ In rice (*Oryza sativa*), expression of aldose reductase increased throughout seed development^57^ and it was also induced by exogenous application of abscisic acid and abiotic stress such as water deficiency and salinity in vegetative tissues.^58^ Other proteins identified from this family included aldo-keto reductases (spots 70, 274, 378, 461, 522, 523, 525, 529, 566 and 567). The aldo-keto reductase family protein is involved in tolerance to abiotic stress like heavy metals and drought^57,59^ and in scavenging reactive oxygen species and their toxic counterparts generated due to environmental stress.^59^ The transient abundance changes of aldose reductase and aldo-keto reductases in dates might suggest a role in fruit development and/or in abiotic stress tolerance.

Heat shock proteins (HSPs) facilitate refolding of denatured proteins in response to high light intensity, heat, hydrogen peroxide and/or pathogenic attack.^60,61^ HSPs also act as molecular chaperones assisting and regulating maturation of target proteins. In this study, three HSP82 (spots 303, 344 and 352) were identified as downregulated at NTR and RIPE, and these were date response specific proteins. The *HSP82* gene is strongly induced by temperatures around 37–42 °C in maize, particularly in embryos and tassels.^62^ In other fruits like peach and apple, differential accumulation of only the small HSPs has been reported after heat stress treatment during ripening and storage.^18,26^ The comparative analysis in this study did not identify small HSPs (molecular mass of 15–30 kDa) but other HSPs including HSP70, also reported in other fruits (Supplementary File 2). Accumulation of nine HSPs (from seven spots: 26, 32, 40, 239, 303, 326 and 352) decreased at NTR and RIPE and two HSP70 (spots 362 and 511) increased transiently at NTR. This result is evidence for the complex role HSPs and suggests that different HSP isoforms might be regulated at various stages during fruit development.

Other stress-related proteins were detected as differentially expressed and included universal-stress protein (USP, spot 660) and E3 ubiquitin ligase (spot 380 and 583) (Supplementary File 2). USP is ubiquitously induced during defense to protect cellular components from heat stress, oxidants or DNA-damaging agents.^63^ In rice, USPs are involved in ethylene-mediated stress adaptation^64^ and it was proposed that they have a similar role in tomato fruit as expression increased simultaneously with ethylene-related enzymes during development and ripening.^19^ In contrast, in dates, USP was downregulated at RIPE, which correlated with the decrease in the ethylene-related enzymes identified. E3 ubiquitin ligase catalyzes the transfer of ubiquitin to target proteins, resulting in their labeling for degradation.^65^ Ubiquitination also plays a crucial role in abiotic and biotic stress responses,^65^ as well as in the regulation of plant immune signaling (for review see, Ref. ^66^). In the current study, E3 ubiquitin ligase (spot 380) accumulation increased at all stages in comparison to S1, while expression of spot 583 decreased at NTR and RIPE. The expected mass of E3 ubiquitin ligase (121 kDa) differed from the observed mass of spots 380 (18 kDa) and 583 (46 kDa), suggesting that these spots might represent either subunits of E3 ubiquitin ligase or truncated version of their full length.^38^

#### Proteins with a role in primary metabolism

Spot abundance of most proteins in the category ‘primary metabolism’ declined from the onset of ripening, and six of them were specific to the date hypanthium. The majority of these proteins have a role in sugar and polysaccharide metabolism (Supplementary File 2). In this category, five spots were identified as sorbitol dehydrogenase (SDH; spots 281, 317, 603, 629 and 635), an enzyme that catalyzes the conversion of sorbitol to fructose, the first step of sorbitol utilization.^67^ In apples, SDH expression was observed throughout fruit development and was proposed to be critical in sugar metabolism during early seed and cortex development.^68^ Rising SDH accumulation was reported during the transition from cell division to cell expansion, and also at ripening in some apple cultivars.^69,70^ Of the five date spots identified as SDH, spot 281 was the most highly upregulated, increasing from 1.83-fold at MD to 11.43-fold at NTR and 23.71-fold at RIPE. Accumulation of spot 603 increased at NTR and RIPE, while the other three spots decreased at all stages (Supplementary File 2). Overall increase in SDH levels is consistent with an increase in fructose and NADH levels during ripening.

A number of UDP-glucose pyrophosphorylase (UGPase) isoforms were also identified as differentially regulated. This enzyme catalyzes the reversible conversion between glucose-1-phosphate and UDP glucose required for sucrose biosynthesis in plants. In banana, transcripts of UGPase increased in response to exogenous ethylene treatment, and this was more severe in the pulp of unripe fruits than in ripe ones,^71^ showing that UGPase expression was ethylene-inducible. Exogenous sucrose and fructose have been reported to increase UGPase abundance in leaves and fruit pulp.^71^ In dates, accumulation of UGPase spots (92, 201 and 508) increased during ripening with the exception of spot 312. This correlated with the increase in SDH levels, and may imply that, similarly to banana, the enzyme is induced by sucrose and fructose but not ethylene since accumulation of two biosynthesis enzymes identified in this study, *S*-adenosylmethionine synthetase (SAMS) and *S*-adenosyl-l-homocysteine hydrolase (SAHH), decreased (Supplementary File 2).

Δ-1-pyrroline-5-carboxylate dehydrogenase (spot 298) and glutamate dehydrogenase (GDH; spot 298), both involved in glutamate biosynthesis, were upregulated at MD and NTR. Δ-1-pyrroline-5-carboxylate dehydrogenase catalyzes the conversion of (*S*)-1-pyrroline-5-carboxylase to *L*-glutamate, and GDH converts *L*-glutamate to glutamate γ-aminobutyric acid.^72^ Previous studies have also depicted a GDH increase in tomato fruits during cell enlargement and a subsequent decline at ripening.^73,74^ Upregulation of GDH andΔ-1-pyrroline-5-carboxylate dehydrogenase was also observed during fruit development in Chinese barberry (*Myrica rubra*).^75^ Like in Chinese barberry, upregulation of both proteins in dates suggests an increase in amino acid metabolism during MD and NTR, the main stages of cell expansion (Figure 3).

Aldehyde dehydrogenase (ALDH) is an enzyme catalyzing the oxidation of various (toxic) aldehydes to their corresponding carboxylic acids in the presence of NAD or NADP as cofactors.^76^ In Arabidopsis, transgenic lines overexpressing *Ath-ALDH3* showed improved tolerance to salt, dehydration, heavy metals and oxidative stress-inducing compounds like H_2_O_2_.^77^ In apple, ALDH was reported as induced by ethylene during fruit development but not at the ripening stage.^69^ In our study, isoforms of ALDH (spots 298, 471 and 554) were upregulated at MD and NTR, and spots 471, 476 and 568 were downregulated at RIPE. The decline of the latter three isoforms of ALDH correlates with the decreased accumulation of ethylene-related proteins, particularly at RIPE, and may suggest a link between ALDH accumulation and the ethylene pathway.^69^

#### Role of ethylene in date ripening

The ethylene biosynthesis pathway starts with the conversion of methionine to *S*-adenosylmethionine (SAM) by SAMS. SAM is then converted to 1-aminocyclopropane-1-carboxylic acid (ACC) by ACC synthase, the rate-limiting step. Finally, ACC is converted to ethylene in the presence of oxygen by ACC oxidase.^78^ SAM can also be demethylated to form *S*-adenosyl-l-homocysteine and catabolized to homocysteine and adenosine by action of SAHH. SAMS has also been linked to the biosynthesis of polyamines that are necessary for cell growth and division,^79,80^ possibly explaining the increased accumulation of SAMS during early fruit development when cell division reaches its maximum. Recent work on papaya reported that increased SAMS and methionine synthase during fruit ripening was a possible indication of their prerequisite for the ethylene burst in climacteric fruits.^36^ In dates, a small peak in ethylene production and respiration rate at early ripening in cultivar ‘Negros’ has been reported,^81,82^ while in another cultivar like ‘Helali’, fruit ripening was inhibited following application of the ethylene inhibitor Ethrel, implying a putative role for ethylene in fruit ripening.^83^ Furthermore, ethylene was detected from 91 DAP in ‘Hillawi’ dates, then increased until Khalal stage before declining sharply at harvest.^11^ However, no change was observed in yellow ‘Barhi’ fruits exposed to 100 ppm ethylene for 48 h at 20 °C and 85%–90% relative humidity.^35^ Expression of SAHH was reported to increase during ripening of climacteric fruits.^84,85^ Results in olive (*Olea europaea*) drupe suggested that decreased expression of SAMS and SAHH is specific to non-climacteric fruits.^80^ In the current study, expression of SAMS (spots 443 and 660) and SAHH (spot 476) was detected as declining at RIPE and this could retard ‘Barhi’ fruit ripening. Although date palm has been classified as a climacteric plant, accumulation patterns of SAMS and SAHH are similar to that of olive drupes, a non-climacteric fruit. In total, about 34 proteins (corresponding to 55 spots; Supplementary File 2) that have been characterized as specific to non-climacteric ripening and only six proteins (corresponding to 17 spots) to climacteric ripening^86^ were identified in the present study, suggesting that cultivar ‘Barhi’ may follow an ethylene-independent ripening process.

#### Proteins associated with secondary metabolism

Proteins with a role in secondary metabolism may also play a pivotal role in environmental stress adaptation.^87^ In this category, 14 proteins were identified and two of them, glutamate 1-semialdehyde aminotransferase (spots 431 and 603) and formamidase-like protein (spots 623 and 635), have not been previously reported in fruits (Table 1). The remaining proteins, including chalcone isomerase isoforms (spots 91, 272, 274 and 374), chalcone synthase (spot 70) and anthocyanidin synthase (spots 69 and 286), have previously been characterized in other fruits. Anthocyanins are pigments commonly induced under stress conditions.^88^ They have also been shown to slow down over-ripening in tomato fruits.^89^ Interestingly, the two spots identified as anthocyanidin synthase, abundance of spot 69 increased at all stages, especially at ripening (21-fold). In contrast, expression of spot 286 declined throughout development, particularly at NTR (−2.44-fold). The marked increase of spot 69 at NTR and RIPE might cause anthocyanidin accumulation and synthesis of anthocyanins.

The correlation between anthocyanins and photosynthesis might be controlled by ethylene since an increase in ethylene was shown to negatively regulate anthocyanin synthesis, while an increase in anthocyanin inhibited photosynthesis.^90^ This suggests that the decrease in ethylene biosynthesis enzymes observed here might have promoted anthocyanin production and indirectly reduced photosynthesis (in correlation with the downregulation of Rubisco ∝-subunit, spots 13 and 14, observed during fruit maturation). Furthermore, increased accumulation of anthocyanins may suggest a response to light stress since their synthesis has also been linked to high light exposure^90^ and protection from photo-oxidative damages.^91,92^ For example, as fruit bearing branches grow heavier and bend downwards away from the protective leaves, direct light exposure to the fruit may increase.

#### Proteins associated with energy production

A total of 20 proteins involved in energy generation were identified. Expression of UMP6 mitochondrial precursor (spot 477), detected as decreasing during ripening, has not previously been detected in other fruits (Table 1). This protein is located in the mitochondria and thylakoid and is involved in organelle organization, DNA metabolic and cell cycle processes. Additionally, nine proteins showed contrasting expression patterns in relation to other fruit species (Table 2). Amongst them, a transaldolase-like protein, transketolase 1, chloroplast oxygen evolving enhancer protein, isoforms of Rubisco α-subunit and malate dehydrogenase (MDH) were identified (Supplementary File 2). The MDH enzyme catalyzes the reversible oxidation of malate to oxaloacetate with concomitant reduction of NAD^+^. It is involved in the TCA cycle and facilitates exchange of metabolites between the cytoplasm and organelles.^93^ In higher plants, four isoforms of MDH were detected, three are NAD-dependent and located in mitochondria, cytoplasm and microbodies, while the fourth one is NADP-dependent and was detected in chloroplasts.^94^ In grapes, expression of MDH increases throughout ripening.^95^ Here, accumulation of spot 421 decreased at all stages, particularly at NTR. Accumulation of spot 482 decreased at MD and then increased at NTR and RIPE, while spot 461 decreased at RIPE. Other spots identified as MDH (spots 129, 137, 274, 412 and 428) were upregulated at different stages of fruit development (Supplementary File 2). Overall significant changes in TCA cycle enzymes suggest increasing energy generation throughout the process of fruit development and ripening and this is consistent with previous findings.^24^

Green fruits contain photosynthetically active chloroplasts that contribute to energy metabolism.^96^ The decline of Rubisco α-subunit isoforms (spots 13 and 14) and chloroplast oxygen evolving enhancer protein (spots 283 and 555) during ripening processes has also been reported in tomatoes,^19^ apricots^27^ and grapes.^24,97^ Reduced expression of the two proteins correlated with a change in skin color from green to light yellow (Figure 3) similarly to apples.^18^ Decrease in Rubisco and chloroplast oxygen evolving enhancer proteins is a potential indicator of chlorophyll degradation, marking the transition of chloroplasts to chromoplasts.^20^ Further, proteins involved in other photosynthesis-related pathways such as the pentose phosphate pathway (PPP) and the Calvin cycle were identified. PPP is an important process that generates Nicotinamide adenine dinucleotide phosphates and pentoses that are the substrates for the Calvin cycle, the dark reactions of photosynthesis. Transketolase 1 is involved in both the non-oxidative phase of PPP and the Calvin cycle. In the former, transketolase 1 is involved in both up- and down-stream of transaldolase-like protein that catalyzes the transfer of a 3-C dihydroxyacetone moiety from sedoheptulose-7-phosphate to glyceraldehyde-3-phosphate. In the Calvin cycle, transketolase 1 converts sedoheptulose-7-phosphate and glyceraldehyde-3-phosphate to aldose ribose-5-phosphate and ketose D-xylulose-5-phosphate.^98^ In the current study, transaldolase-like protein (spot 577) and transketolase 1 (spot 202) significantly decreased at all stages, and more prominently so at NTR and RIPE, suggesting a downregulation of non-oxidative phase of PPP and the Calvin cycle.

The reduction of proteins associated with photosynthesis correlated with the reduction of pyruvate dehydrogenase (PDH) E1 component (spots 2 and 506) and the increase of mitochondrial pyruvate dehydrogenase kinase isoform 1 (mPDK, spot 431, also classified as a signaling protein). PDH E1 component is a key enzyme involved in the decarboxylation of pyruvate and reductive acetylation of lipoic acid to form acetyl-CoA, while mPDK regulates activity of the PDH complex by phosphorylating PDH E1 component.^99^ In grapes, the mPDK isoform was induced in a ripening-specific manner.^100^ In the current study, decrease in PDH E1 component and increase in mPDK accumulation both occurred in a ripening-specific manner (i.e., at NTR and RIPE). This may indicate that while accumulation of PDH E1 component decreased, the increased abundance of mPDK could promote inactivation of the remaining PDH E1 components of PDH complex.

Enzymes involved in the glycolysis pathway were also identified including fructose-bisphosphate aldolase (spot 566), triosephosphate isomerase (spot 503), phosphoglycerate kinase (spot 431), phosphoglycerate mutase (spot 531) and enolase (spot 18). Triosephosphate isomerase was upregulated at all stages, and similarly to apples, possibly enhancing respiration and catabolic metabolism.^18^ Expression of phosphoglycerate kinase increased at NTR and RIPE, and phosphoglycerate mutase at RIPE, while that of enolase decreased at NTR and RIPE. Increases in phosphoglycerate kinase and phosphoglycerate mutase suggest enhanced energy production, particularly since this correlated with the upregulation of ATP synthase β-chain (spot 312, 419 and 507) of the ATP biosynthesis pathway. ATP synthase β-chain is involved with fuelling cellular energy, and has recently been demonstrated as a key regulator of fruit ripening and senescence.^101^ In litchi (*Litchi chinensis*) fruit pericarp, ATP synthase β-chain accumulation significantly increased during fruit development and early post-harvest, while alternative oxidase 1 significantly increased two days post-harvest.^101^ Exogenous application of ATP induced a reduction of alternative oxidase 1 expression, enhancing antioxidant systems and delayed pericarp browning in litchi^101–103^ and ‘Conference’ pears.^104^ Additionally, elevated ATP and energy levels decrease membrane permeability and production of reactive oxygen species during ripening.^104,105^ Here, expression of ATP synthase β-chain, spot 507, increased at RIPE (9.99-fold), spot 419 transiently increased at NTR (2.78-fold), while spot 312 decreased at NTR (−2.28-fold) and RIPE (−1.65-fold, Supplementary File 2). The overall increase of this ATP-generating enzyme particularly at NTR and RIPE could help maintain membrane integrity and promote antioxidant capacity, which could in turn protect dates against oxidative stress and senescence.^102,103^

#### Proteins implicated in cellular structures

Decline in fruit firmness during ripening and senescence is partially controlled by changes in the expression of cytoskeleton-related proteins and cell wall-degrading enzymes.^38,42^ These can lead to biochemical and physical/structural alterations in cells and their walls. Of the proteins identified in this category, actin (spot 317), fibrillin (spot 3 and 53), plastid-lipid associated protein 3 (spot 19) and tubulin β-chain (spot 356) showed contrasting differential accumulation in dates compared to other fruits such as grapes (Table 2 and Supplementary File 2). Actins are involved in cytoplasmic streaming, cell division, organelle movement and cell shape maintenance and, in grapes, their abundance remained elevated throughout fruit development.^22,106^ In this study, actin was identified in four spots (129, 317, 400 and 487). Spot 129 was upregulated at NTR, spot 400 increased at MD and NTR, and spot 487 increased at NTR and RIPE, while spot 317 decreased at RIPE (Table 2 and Supplementary File 2). Besides actins, tubulins, which are cytoskeletal proteins implicated in cellular processes including the preservation of cell structure and intracellular transport, were also identified. Increased tubulin levels have been reported in the early phase of cucumber (*Cucumis sativus*) development^107^ and elevated expression of actins and tubulins has been observed during rapid cell enlargement, a phase characterized by extensive cytoskeleton rearrangement.^23^ Here, eight spots (14, 56, 189, 197, 356, 359, 417 and 422) identified as tubulins were downregulated at NTR and RIPE, with the exception of spot 356 that increased at RIPE. Similarly to the present study, expression of tubulins was reported to decline during grape berry development and was very limited during ripening. Additionally, xylose isomerase (spot 507 and 508), which is involved in a reversible conversion of D-xylose to xylulose, was upregulated at ripening. D-xylose is the primary constituent of xylans, which are involved in cell wall stabilization.^108^

#### Posttranslational modifications

The current study highlights interactions of various cellular and metabolic processes linked to fruit development and ripening, as observed by changes in protein accumulation. A number of proteins undergo PTMs for acquiring stability, subcellular localization, (de)-activation and/or protein–protein interactions. Previous studies reported carbonylation of several proteins during senescence in apple^109^ and peach, ^110^ initiating PTM investigations in fruit proteomics. In the present study, a total of 362 methionine oxidation sites positioned on 204 proteins and 443 phosphosites on 328 proteins were detected using Scaffold PTM (Supplementary File 5). These PTMs potentially have important roles in regulating fruit development and ripening and merit a detailed follow-up study.

## Conclusions

Here, we provide the first detailed comparative proteome of the date fruit, a fruit that is of particular horticultural interest in the Middle East and North Africa. Proteomics afforded four major insights in differential protein expression during fruit development and ripening. The first is an increase in the accumulation of abiotic stress-responsive proteins during ripening with the exception of HSPs and universal stress protein, which decreased, suggesting intricate metabolic systems for stress response and, in particular, the ability to withstand high temperatures. The second is an overall increase of glycolysis and TCA cycle-related proteins in contrast to pentose phosphate and photosynthesis from ripening onset. This indicates a shift in energy supply between the fruit development stages markedly S1-MD and NTR-RIPE. The third is a decrease of two enzymes involved in ethylene biosynthesis, *S*-adenosylmethionine synthetase and *S*-adenosyl-l-homocysteine hydrolase, which are among the 34 identified proteins proposed to be specific to non-climacteric ripening. This implies that the cultivar ‘Barhi’ might follow an ethylene-independent ripening process. The fourth is a differential accumulation of enzymes involved in secondary metabolism, notably the sharp increase in anthocyanidin synthase, from the anthocyanin biosynthesis pathway, during ripening. Anthocyanins act as natural antioxidants and play a crucial protective role in response to light. The results from this investigation will further our understanding of the role and regulation of proteins and pathways during fruit ripening, and will contribute to the development of innovative practices for fruit quality improvements.

## Figures and Tables

**Table 1:** Date specific proteins identified as differentially regulated during fruit development and ripening

Spot no.	Identified protein	Protein probability	Accession no.	Theoretical MW (Da)/p*I*	Observed MW (Da)/p*I*	Sequence coverage	NUP	FC S1/MD	FC S1/NTR	FC S1/R
01	Metabolism									
197	Furostanol glycoside 26-*O*-β-glucosidase	100.00%	30s65509133g003	64132/5.34	61000/6.1	13.30%	7	ns	ns	−7.28
227	Dolichyl-phosphate-mannose-glycolipid α-mannosyltransferase	98.50%	30s785981g002	64116/9.91	39000/5.6	1.44%	1	ns	4.20	6.00
235	Lysosomal α-subunit	100.00%	30s945081g001	202855/7.02	82000/5.35	1.44%	3	ns	−3.61	1.99
385	Disproportionating enzyme^a^	99.70%	30s1194551g005	52152/5.41	61000/5.0	3.70%	1	ns	ns	−1.91
482	AMP-dependent	100.00%	30s993441g001	29388/5.16	66000/4.5	12.50%	3	−2.02	4.57	5.56
566	Ornithine carbamoyltransferase^a^	99.50%	30s699641g001	42893/7.66	16000/4.3	3.08%	1	ns	−2.03	−2.99
02	Energy									
477	UMP6_mitochondrial flags: precursor	100.00%	30s1009951g003	16907/5.11	15000/5.95	13.60%	2	ns	−2.16	−2.70
03	Cell growth/division									
366	Growth regulator	98.10%	30s797691g002	19927/10.01	62000/5.6	6.29%	1	ns	ns	−3.21
387	KU P80 DNA	99.50%	30s862811g001	75649/6.15	20000/4.9	1.48%	1	2.75	ns	ns
04	Transcription									
302	Gata transcription factor 25	99.70%	30s1133641g001	80728/9.32	25000/5.65	1.37%	1	ns	2.14	−1.96
302	Inducer of cbf expression 1 DNA binding transcription activator transcription factor	99.70%	30s953031g001	38914/10.25	25000/5.65	2.51%	1	ns	2.14	−1.96
05	Protein synthesis									
249	Gag-pol polyprotein	99.70%	30s811431g002	49976/7.27	24000/4.5	2.04%	1	ns	1.65	1.72
274	Aspartyl-tRNA synthetase^a^	100.00%	30s6550950g017	66976/5.84	65000/4.75	19.30%	7	ns	ns	3.30
06	Protein destination and storage									
129	Proline iminopeptidase^a^	100.00%	30s775611g009	40654/5.64	31000/5.45	4.57%	2	ns	2.22	ns
210	Proteasome subunit β type 7-A	100.00%	30s1126271g001	29520/6.79	19000/4.4	4.40%	1	−2.20	−4.21	−4.79
300	Nuclear transport	99.20%	30s1197741g004	16883/5.77	11000/5.25	6.80%	1	−2.91	−3.50	−1.66
370	GDP dissociation inhibitor^a^	100.00%	30s1179061g005	49697/5.83	47000/4.8	4.50%	2	ns	1.74	2.60
531	Chaperonin containing t-complex protein epsilon	100.00%	30s1179991g005	59114/5.67	30000/6.15	7.32%	4	ns	ns	1.64
08	Intracellular traffic									
417	Protein binding structural molecule	99.90%	30s784571g001	33386/5.26	50000/5.75	6.89%	1	ns	−1.93	−2.51
11	Disease/defence									
303	Heat shock protein 82	100.00%	30s947641g003	80204/5.04	82000/5.9	10.70%	2	ns	−2.50	−1.57
303	Heat shock protein 82	100.00%	30s722381g008	89708/5.18	82000/5.9	12.40%	3	ns	−2.50	−1.57
344	Heat shock protein 82	99.60%	30s1082281g001, 30s722381g008, 30s947641g003	89708/4.66	80000/5.85	3.17%	1	ns	−2.73	−1.57
352	Heat shock protein 82	99.70%	30s1082281g001, 30s722381g008, 30s947641g003	89708/4.66	54000/5.9	3.17%	1	ns	ns	−2.00
401	Aldose reductase	100.00%	30s1006721g002	34679/6.01	19000/5.0	14.10%	5	2.13	ns	1.78
12	Unclear classification									
91	Riboflavin α- subunit	99.00%	30s703111g002	28760/9.09	19000/5.5	5.22%	2	2.09	−1.58	−2.61
199	Nucleic acid binding	99.80%	30s1050381g022	36142/4.99	22000/5.88	6.98%	1	ns	−2.95	−2.56
477	Nucleic acid binding	100.00%	30s1050381g022	14374/4.99	15000/5.95	10.90%	2	ns	−2.16	−2.70
507	JHL06P13.3-like protein^b^	99.80%	30s1042771g010	50733/5.46	43000/5.3	2.22%	1	ns	3.96	9.99
508	JHL06P13.3-like protein^b^	100.00%	30s1042771g010	50733/5.46	55000/6.26	4.44%	2	ns	1.73	ns
561	Mitochondrial glycoprotein	99.20%	30s796661g001	33383/5.55	26000/4.35	4.05%	1	ns	ns	−2.03
20	Secondary metabolism									
431	Glutamate 1-semialdehyde aminotransferase	100.00%	30s1039641g001	50533/5.98	39000/4.8	5.29%	2	ns	4.54	5.18
603	Glutamate 1-semialdehyde aminotransferase	99.60%	30s1039641g001	50533/5.98	55000/5.33	2.54%	1	ns	4.07	3.70
623	Formamidase-like protein	99.50%	30s702741g005	48663/5.73	34000/4.5	2.47%	1	ns	−1.73	−3.02
635	Formamidase-like protein	99.50%	30s702741g005	48663/5.73	54000/5.1	2.47%	1	−1.66	−3.63	−4.27

Abbreviations: ns, not significant; NUP, no. of unique peptides.

a Proteins identified in other fruit studies but not shown to be linked with fruit development or ripening.

b Highly homologous to Barbados nut (*Jatropha curcas*) and involved in ATP-binding in addition to having ATP-dependent helicase activity.

**Table 2:** Differentially regulated proteins in the date hypanthium showing contrasting transient patterns compared to other fruits

Spot no.	Identified protein	Protein probability	Accession no.	Theoretical MW (Da)/p*I*	Observed MW (Da) /p*I*	Sequence coverage	NUP	FC S1/MD	FC S1/NTR		FC S1/R	
01	Metabolism											
91	Isoamyl acetate-hydrolyzing esterase	100.00%	30s696051g001	36883/5.86	19000/5.5	4.79%	2	2.09	−1.58		−2.61	
192	Glucan endo-1,4-β-glucosidase	100.00%	30s896091g001	16069/6.54	60000/6.1	14.50%	3	−1.90	−2.96		−15.84	
196	Cytochrome p450	99.70%	30s6550951g018	24174/4.39	23000/6.1	7.37%	1	ns	ns		−2.09	
202	Transketolase 1	99.00%	30s656601g005	84589/5.66	95000/4.9	1.42%	1	1.86	−8.33		−8.45	
312	UDP-glucose pyrophosphorylase	100.00%	30s904561g011	34207/6.1	54000/5.25	7.47%	2	ns	−2.28		−1.65	
317	Sorbitol dehydrogenase	100.00%	30s830031g004	38361/5.53	45000/5.6	21.30%	7	ns	ns		−1.56	
443	*S*-adenosylmethionine synthetase	100.00%	30s1183641g001	43368/5.68	43000/5.1	11.20%	3	ns	−2.25		−4.48	
443	*S*-adenosylmethionine synthetase	100.00%	30s1207141g006	43344/5.76	43000/5.1	8.08%	1	ns	−2.25		−4.48	
471	Aldehyde dehydrogenase	100.00%	30s693451g001	59067/6.48	55000/4.65	18.80%	11	ns	2.18		−2.17	
476	*S*-adenosyl-l-homocysteine hydrolase	100.00%	30s1132381g003, 30s825931g001	38838/6.72	45000/6.0	13.00%	6	ns	ns		−2.83	
476	Aldehyde dehydrogenase	100.00%	30s693451g001	59067/6.98	45000/6.0	12.20%	5	ns	ns		−2.83	
522	Reversibly glycosylated polypeptide	100.00%	30s969461g002	41051/6.12	37000/5.9	3.59%	2	ns	−2.27		−3.20	
573	Pyruvate decarboxylase	99.70%	30s919171g003	38883/8.66	60000/6.2	3.46%	1	ns	−2.33		−5.94	
660	*S*-adenosylmethionine synthetase	99.60%	30s1183641g001, 30s1207141g006, 30s799761g004, 30s933141g001	43344/5.68	25000/6.1	3.28%	1	ns	ns		−2.64	
02	Energy											
2	Pyruvate dehydrogenase E1 component β-subunit	99.90%	30s852391g002	40148/5.46	34000/5.74	6.43%	1	ns	−3.12		−2.73	
18	Enolase	100.00%	30s663761g002	47828/5.91	55000/5.15	7.64%	3	ns	−1.90		−1.50	
403	Citrate synthase	99.50%	30s1040901g002	30711/6.25	55000/4.3	6.64%	1	ns	2.52		3.23	
421	Malate dehydrogenase	100.00%	30s892681g001	48314/7.11	49000/6.25	6.11%	1	−1.64	−7.02		−4.54	
461	Malate dehydrogenase	100.00%	30s903851g004, 30s946431g012	26083/8.76	30000/4.7	4.39%	2	ns	ns		−2.09	
506	Pyruvate dehydrogenase E1 component β-subunit	100.00%	30s855801g002	22740/5.7	35000/5.31	13.00%	2	ns	−1.60		−1.70	
531	Bisphosphoglycerate-independent phosphoglycerate mutase	100.00%	30s661041g002, 30s693071g003	43543/5.55	30000/6.15	5.60%	1	ns	ns		1.64	
566	Fructose-bisphosphate aldolase	100.00%	30s1148281g010	40222/7.55	16000/4.4	12.10%	4	ns	−2.03		−2.99	
577	Transaldolase-like protein	100.00%	30s1065691g004	43557/5.05	55000/6.5	4.88%	2	−1.79	−7.48		−4.49	
03	Cell growth/division											
45	Condensin complex subunit 1	99.30%	30s702551g004	127265/5.78	51000/5.34	1.47%	1	ns	2.53		1.95	
252	Enhancer of polycomb-like protein	99.70%	30s827291g002	51710/9.99	18000/6.2	5.79%	1	4.51	4.99		2.11	
415	14-3-3-like protein	99.70%	30s819041g002	17032/5.09	19000/6.24	7.95%	1	2.71	2.55		1.72	
04	Transcription											
416	Transcription factor IIA small subunit	99.70%	30s656261g002, 30s760301g006	12093/6.08	27000/4.7	10.40%	1	2.52	3.51		2.46	
05	Protein synthesis											
45	Translation initiation factor (eif-4a)	99.90%	30s724051g001, 30s997411g005	47064/5.39	51000/5.34	2.42%	1	ns	2.53		1.95	
53	Elongation factor 1	99.20%	30s667161g002	16163/4.26	21000/6.6	11.90%	1	−1.84	−1.79		1.55	
287	30s ribosomal protein s1	97.70%	30s783011g009	45242/4.95	35000/6.25	2.15%	1	1.58	8.73		11.49	
416	Peptide chain release factor, putative	99.70%	30s682511g003	9163/4.85	27000/4.7	13.30%	1	2.52	3.51		2.46	
504	60s ribosomal protein l23a	99.70%	30s1035671g001	9453/11.22	88000/6.0	11.00%	1	1.62	ns		−3.63	
525	Translation initiation factor (eif-4a)	100.00%	30s724051g001, 30s997411g005	47064/5.39	39000/5.34	9.93%	4	ns	3.98		5.12	
06	Protein destination and storage											
3	Cysteine protease	98.80%	30s790241g001	36042/4.87	22000/6.55	4.98%	1	ns	−2.48		−1.80	
32	Luminal binding protein	100.00%	30s685511g001	56109/8.42	82000/5.95	7.24%	2	ns	ns		−2.52	
92	Mitochondrial processing peptidase	100.00%	30s927641g002	59451/5.76	55000/5.48	6.08%	3	ns	1.92		1.88	
239	Luminal binding protein	100.00%	30s685511g001	56109/8.42	70000/5.8	18.00%	8	ns	ns		−1.62	
288	Cysteine protease	99.70%	30s790241g001	36042/4.87	32000/6.3	4.98%	1	ns	2.43		2.24	
303	Subtilisin-like serine proteinase	100.00%	30s808251g002	48672/4.79	82000/5.9	5.76%	2	ns	−2.50		−1.57	
419	Mitochondrial processing peptidase	100.00%	30s927641g002	59451/5.76	55000/5.5	4.60%	1	ns	2.78		ns	
609	Cysteine protease	99.60%	30s790241g001	36042/4.87	31000/6.48	4.98%	1	ns	−1.86		−1.99	
611	Cysteine protease	99.40%	30s790241g001	36042/4.87	29000/5.15	4.98%	1	ns	ns		−2.35	
07	Transporters											
99	Vacuolar ATP synthase subunit v- proton pump b v-ATPase 57 kDa	100.00%	30s735571g002, 30s837971g002	58290/5.43	58000/5.9	3.08%	2	ns	2.13		ns	
312	ATP synthase β chain	100.00%	30s884401g004	55268/5.31	54000/5.25	5.10%	2	ns	−2.28		−1.65	
419	ATP synthase β chain	100.00%	30s884401g004	55268/5.31	55000/5.5	7.25%	3	ns	2.78		ns	
507	ATP synthase β chain	99.50%	30s884401g004	55268/5.31	43000/5.3	2.75%	1	ns	3.96		9.99	
403	CMP-sialic acid	99.90%	30s808171g003	23646/9.71	55000/4.3	9.52%	1	ns	2.52		3.23	
468	Mitochondrial deoxynucleotide carrier, putative	99.70%	30s656531g003	36808/9.09	16000/5.35	2.69%	1	ns	−2.26		−2.47	
09	Cell structure											
3	Fibrillin-like protein	98.80%	30s724791g001, 30s837971g003	39091/9.45	22000/6.55	4.19%	1	ns	−2.48		−1.80	
19	Plastid-lipid associated protein 3	98.30%	30s761251g002	20316/6.28	37000/6.8	5.35%	1	ns	−1.61		−2.37	
53	Fibrillin-like protein	99.20%	30s724791g001, 30s837971g003	39091/9.45	21000/6.6	4.19%	1	−1.84	−1.79		1.55	
317	Actin	100.00%	30s1070141g012, 30s717671g011, 30s844721g006, 30s951221g005	41610/5.31	45000/5.6	22.30%	3	ns	ns		−1.56	
356	Tubulin β-2	100.00%	30s837411g003, 30s965611g001	50234/4.72	34000/5.2	9.60%	1	ns	ns		2.39	
356	Tubulin β-chain	100.00%	30s1088541g002, 30s697331g005, 30s708731g001	50095/4.77	34000/5.2	9.60%	1	ns	ns		2.39	
10	Signal transduction											
34	Germin-like protein	98.10%	30s1014231g001	13337/5.63	18000/4.5	17.60%	1	2.71		5.01		4.50
96	Nucleoside diphosphate kinase	100.00%	30s835621g002	12145/9.0	16000/4.9	28.80%	3	1.74		−1.50		−1.62
314	IN2-1 protein	99.80%	30s696261g003	37485/6.46	19000/6.0	2.73%	1	−1.83		ns		6.10
587	Zinc finger	100.00%	30s796661g001	41956/5.55	35000/5.8	5.15%	2	−1.85		−8.66		−6.63
11	Disease/defence											
13	Leucine rich repeat-containing	100.00%	30s857351g006	49345/4.81	64000/6	9.64%	3	ns		ns		−1.95
32	Heat shock protein 70	100.00%	30s941391g004	71057/5.13	82000/5.95	10.90%	3	ns		ns		−2.52
81	Glutathione transferase	100.00%	30s806101g003	37154/5.57	30000/5.4	8.00%	2	ns		ns		1.67
239	Heat shock protein 70	100.00%	30s795061g002	71222/5.17	70000/5.8	19.40%	1	ns		ns		−1.62
239	Heat shock protein 70	100.00%	30s941391g005	61596/5.11	70000/5.8	18.10%	3	ns		ns		−1.62
239	Heat shock protein 70	100.00%	30s941391g004	71057/5.13	70000/5.8	15.10%	1	ns		ns		−1.62
239	Heat shock cognate 70 kDa expressed	99.80%	30s1034811g003	13813/4.45	70000/5.8	17.60%	1	ns		ns		−1.62
246	ABA-hypersensitive germination 2 nucleic acid binding ribonuclease	98.70%	30s1120391g003	79218/5.98	22000/6.37	1.44%	1	ns		−1.50		−2.15
274	Aldo/keto reductase	100.00%	30s1183861g001	38732/5.97	65000/4.75	10.50%	3	ns		ns		3.30
357	ATGLX1 (glyoxalase i homolog)	99.70%	30s1166451g001	16766/5.94	24000/5.78	8.97%	1	ns		1.75		1.91
362	Heat shock protein 70	100.00%	30s941391g005	61596/5.11	70000/5.9	5.90%	2	ns		1.83		−3.04
362	Heat shock protein 70	100.00%	30s795061g002	71222/5.17	70000/5.9	6.02%	3	ns		1.83		−3.04
378	Aldo/keto reductase	99.80%	30s1183861g001	38732/5.97	65000/4.85	2.27%	1	ns		ns		3.09
403	Catalase	100.00%	30s1080231g005	55425/6.62	55000/4.3	10.70%	5	ns		2.52		3.23
404	Catalase	100.00%	30s1080231g005	55425/6.62	55000/4.25	4.62%	2	ns		3.79		3.82
503	Glutathione s-transferase GSTF2	100.00%	30s987821g001	13597/6.89	19000/4.7	14.90%	2	2.27		2.55		2.52
511	Heat shock protein 70	99.80%	30s941391g005	61596/5.11	70000/5.85	3.76%	1	ns		2.18		−2.14
523	Aldo/keto reductase	100.00%	30s1183861g001	38732/5.97	43000/5.45	11.90%	3	ns		1.84		2.64
525	Aldo/keto reductase	100.00%	30s1183861g001	38732/5.97	39000/5.34	9.07%	2	ns		3.98		5.12
583	E3 ubiquitin ligase	99.70%	30s1065691g004	121028/6.79	46000/6.25	0.91%	1	ns		−5.11		−6.36
20	Secondary metabolism											
63	Isopentenyl diphosphate isomerase	100.00%	30s802901g001	32219/7.16	20000/5.8	6.86%	2	1.75		ns		−2.50
63	Isopentenyl pyrophosphate isomerase	100.00%	30s762371g001	17552/5.91	20000/5.8	13.10%	2	1.75		ns		−2.50
91	Chalcone isomerase	100.00%	30s1070141g002	24742/5.29	19000/5.5	4.27%	1	2.09		−1.58		−2.61
272	Chalcone isomerase	100.00%	30s1070141g002	24742/5.29	20000/5.7	24.80%	6	ns		−5.09		−4.71
274	Chalcone isomerase	100.00%	30s1070141g002	24742/5.29	65000/4.75	14.50%	3	ns		ns		3.30
286	Anthocyanidin synthase	100.00%	30s1125611g001	38960/5.99	23000/4.7	5.51%	4	−1.63		−2.44		−1.62

Abbreviations: ns, not significant; NUP, no. of unique peptides.
