# Supplementary material for: Dynamic changes in the date palm fruit proteome during development and ripening
Source: Hortic Res. 2014 Aug 6;1:14039–. doi: 10.1038/hortres.2014.39 (PMC4596323; doi:10.1038/hortres.2014.39)
Supplement: Supplementary File 2 [file hortres201439-s1.doc]

**Supplementary file 2. Proteins differentially expressed in the date hypanthium during development and ripening.**

| **Spot no.** | **Identified protein** | **Protein probability** | **Accession no.** | | | | **Theoretical MW (Da)** | **Observed MW (Da)** | | **Sequence coverage** | | **NUP** | | | **FC S1/MD** | | | **FC S1/NTR** | | **FC S1/R** | |
| --- | --- | --- | --- | --- | --- | --- | --- | --- | --- | --- | --- | --- | --- | --- | --- | --- | --- | --- | --- | --- | --- |
| **01** | **Metabolism** |  |  | | | |  |  | |  | |  | | |  | | |  | |  | |
| 43 | Phenylalanine ammonia-lyase | 100.00% | 30s1062051g002 | | | | 82565 | 67000 | | 2.94% | | 2 | | | 1.54 | | | 2.99 | | 4.10 | |
| 88 | Glucan endo-1,4-β-glucosidase | 100.00% | 30s896091g001 | | | | 16069 | 25000 | | 14.50% | | 3 | | | ns | | | ns | | 4.99 | |
| 91 | Isoamyl acetate-hydrolyzing esterase | 100.00% | 30s696051g001 | | | | 36883 | 19000 | | 4.79% | | 2 | | | 2.09 | | | -1.58 | | -2.61 | |
| 92**^nc^** | UDP-glucose pyrophosphorylase | 100.00% | 30s904561g011 | | | | 34207 | 55000 | | 8.12% | | 2 | | | ns | | | 1.92 | | 1.88 | |
| 182 | Glucan endo-1,4-β-glucosidase | 99.70% | 30s896091g001 | | | | 16069 | 29000 | | 5.92% | | 1 | | | ns | | | 2.40 | | 1.70 | |
| 192 | Glucan endo-1,4-β-glucosidase | 100.00% | 30s896091g001 | | | | 16069 | 60000 | | 14.50% | | 3 | | | -1.90 | | | -2.96 | | -15.84 | |
| 196**^nc^** | Cytochrome p450 | 99.70% | 30s6550951g018 | | | | 24174 | 23000 | | 7.37% | | 1 | | | ns | | | ns | | -2.09 | |
| 197 | Furostanol glycoside 26-o-β-glucosidase | 100.00% | 30s65509133g003 | | | | 64132 | 61000 | | 13.30% | | 7 | | | ns | | | ns | | -7.28 | |
| 201**^nc^** | UDP-glucose pyrophosphorylase | 99.50% | 30s904561g011 | | | | 16069 | 60000 | | 5.19% | | 1 | | | ns | | | 1.53 | | 1.79 | |
| 227 | Dolichyl-phosphate-mannose-glycolipid α-mannosyltransferase | 98.50% | 30s785981g002 | | | | 64116 | 39000 | | 1.44% | | 1 | | | ns | | | 4.20 | | 6.00 | |
| 228**^nc^** | Glycerol-3-phosphate dehydrogenase | 97.10% | 30s1093671g002 | | | | 9314 | 80000 | | 11.40% | | 1 | | | ns | | | ns | | 2.17 | |
| 235 | Lysosomal α-subunit | 100.00% | 30s945081g001 | | | | 202855 | 82000 | | 1.44% | | 3 | | | ns | | | -3.61 | | 1.99 | |
| 274 | NADP-dependent malic enzyme | 100.00% | 30s669671g001 | | | | 61993 | 65000 | | 5.40% | | 2 | | | ns | | | ns | | 3.30 | |
| 281 | Sorbitol dehydrogenase | 100.00% | 30s830031g004 | | | | 38361 | 39000 | | 9.70% | | 2 | | | 1.83 | | | 11.43 | | 23.71 | |
| 298**^nc^** | Glutamate decarboxylase | 100.00% | 30s656741g003 | | | | 55946 | 55000 | | 14.90% | | 6 | | | 1.52 | | | 4.55 | | ns | |
| 298 | Delta-1-pyrroline-5-carboxylate dehydrogenase | 100.00% | 30s1207061g003 | | | | 57177 | 55000 | | 5.28% | | 3 | | | 1.52 | | | 4.55 | | ns | |
| 298**^nc^** | Aldehyde dehydrogenase | 100.00% | 30s982851g002 | | | | 54459 | 55000 | | 5.82% | | 1 | | | 1.52 | | | 4.55 | | ns | |
| 312**^nc^** | UDP-glucose pyrophosphorylase | 100.00% | 30s904561g011 | | | | 34207 | 54000 | | 7.47% | | 2 | | | ns | | | -2.28 | | -1.65 | |
| 317 | Sorbitol dehydrogenase | 100.00% | 30s830031g004 | | | | 38361 | 45000 | | 21.30% | | 7 | | | ns | | | ns | | -1.56 | |
| 378 | Phenylalanine ammonia lyase | 99.60% | 30s1062051g002 | | | | 82565 | 65000 | | 1.74% | | 1 | | | ns | | | ns | | 3.09 | |
| 385 | Disproportionating enzyme | 99.70% | 30s1194551g005 | | | | 52152 | 61000 | | 3.70% | | 1 | | | ns | | | ns | | -1.91 | |
| 443**^nc^** | S-adenosylmethionine synthetase | 100.00% | 30s1183641g001 | | | | 43368 | 43000 | | 11.20% | | 3 | | | ns | | | -2.25 | | -4.48 | |
| 443**^nc^** | S-adenosylmethionine synthetase | 100.00% | 30s1207141g006 | | | | 43344 | 43000 | | 8.08% | | 1 | | | ns | | | -2.25 | | -4.48 | |
| 471**^nc^** | Aldehyde dehydrogenase | 100.00% | 30s693451g001 | | | | 59067 | 55000 | | 18.80% | | 11 | | | ns | | | 2.18 | | -2.17 | |
| 471 | Adenosylhomocysteinase S-adenosyl-L-homocysteine hydrolase | 100.00% | 30s825931g001 | | | | 35583 | 55000 | | 13.00% | | 6 | | | ns | | | 2.18 | | -2.17 | |
| 476 | S-adenosyl-L-homocysteine hydrolase | 99.90% | 30s1132381g003, 30s825931g001 | | | | 38838 | 45000 | | 3.67% | | 1 | | | ns | | | ns | | -2.83 | |
| 476**^nc^** | Aldehyde dehydrogenase | 100.00% | 30s693451g001 | | | | 59067 | 45000 | | 12.20% | | 5 | | | ns | | | ns | | -2.83 | |
| 482 | AMP-dependent synthetase | 100.00% | 30s993441g001 | | | | 29388 | 66000 | | 12.50% | | 3 | | | -2.02 | | | 4.57 | | 5.56 | |
| 507**^cl^** | Xylose isomerase | 100.00% | 30s1161861g002 | | | | 53732 | 78000 | | 11.00% | | 5 | | | ns | | | 3.96 | | 9.99 | |
| 508**^nc^** | UDP-glucose pyrophosphorylase | 100.00% | 30s904561g011 | | | | 34207 | 55000 | | 5.19% | | 1 | | | ns | | | 1.73 | | ns | |
| 508**^cl^** | Xylose isomerase | 100.00% | 30s1161861g002 | | | | 53732 | 55000 | | 10.80% | | 4 | | | ns | | | 1.73 | | ns | |
| 522 | Reversibly glycosylated polypeptide | 100.00% | 30s969461g002 | | | | 41051 | 37000 | | 3.59% | | 2 | | | ns | | | -2.27 | | -3.20 | |
| 532**^nc^** | Bis (5-adenosyl)-triphosphatase | 98.50% | 30s693071g005 | | | | 15101 | 17000 | | 7.69% | | 1 | | | -1.96 | | | -2.80 | | -4.66 | |
| 551 | Pyruvate decarboxylase | 99.50% | 30s660411g001 | | | | 38883 | 55000 | | 3.46% | | 1 | | | ns | | | 1.94 | | 1.53 | |
| 554**^nc^** | Aldehyde dehydrogenase | 99.50% | 30s982851g002 | | | | 54459 | 15000 | | 3.01% | | 1 | | | 4.35 | | | ns | | ns | |
| 566**^nc^** | Ornithine carbamoyltransferase | 99.50% | 30s699641g001 | | | | 42893 | 16000 | | 3.08% | | 1 | | | ns | | | -2.03 | | -2.99 | |
| 573 | Pyruvate decarboxylase | 99.70% | 30s919171g003 | | | | 38883 | 60000 | | 3.46% | | 1 | | | ns | | | -2.33 | | -5.94 | |
| 603 | Sorbitol dehydrogenase | 99.60% | 30s830031g004 | | | | 38361 | 55000 | | 3.32% | | 1 | | | ns | | | 4.07 | | 3.70 | |
| 612 | 2-oxoglutarate-dependent dioxygenase | 100.00% | 30s767941g003 | | | | 35326 | 39000 | | 3.18% | | 1 | | | ns | | | 3.99 | | 8.43 | |
| 629 | Sorbitol dehydrogenase | 98.50% | 30s830031g004 | | | | 38361 | 21000 | | 3.32% | | 1 | | | -2.12 | | | ns | | -1.68 | |
| 635 | Sorbitol dehydrogenase | 100.00% | 30s830031g004 | | | | 38361 | 54000 | | 5.82% | | 2 | | | -1.66 | | | -3.63 | | -4.27 | |
| 660**^nc^** | S-adenosylmethionine synthetase | 99.60% | 30s1183641g001, 30s1207141g006, 30s799761g004, 30s933141g001 | | | | 43344 | 25000 | | 3.28% | | 1 | | | ns | | | ns | | -2.64 | |
| **02** | **Energy** |  |  | | | |  |  | |  | |  | | |  | | |  | |  | |
| 2 | Pyruvate dehydrogenase E1 component β-subunit | 99.90% | 30s852391g002 | | | | 40148 | 34000 | | 6.43% | | 1 | | | ns | | | -3.12 | | -2.73 | |
| 13 | Rubisco subunit binding-protein α- subunit | 100.00% | 30s1048241g003 | | | | 39658 | 64000 | | 10.90% | | 2 | | | ns | | | ns | | -1.95 | |
| 13 | Rubisco subunit binding-protein α- subunit | 100.00% | 30s1205991g001 | | | | 24594 | 64000 | | 25.00% | | 3 | | | ns | | | ns | | -1.95 | |
| 13 | Rubisco subunit binding-protein α- subunit | 99.90% | 30s655141g001 | | | | 61111 | 64000 | | 8.81% | | 1 | | | ns | | | ns | | -1.95 | |
| 14 | Rubisco subunit binding-protein α- subunit | 99.90% | 30s1205991g001 | | | | 24594 | 57000 | | 12.30% | | 1 | | | ns | | | -2.38 | | -2.52 | |
| 18 | Enolase | 100.00% | 30s663761g002 | | | | 47828 | 55000 | | 7.64% | | 3 | | | ns | | | -1.90 | | -1.50 | |
| 91**^cl^** | Oxygen-evolving enhancer protein chloroplast | 100.00% | 30s919171g003 | | | | 35305 | 19000 | | 14.40% | | 5 | | | 2.09 | | | -1.58 | | -2.61 | |
| 129 | NAD-malate dehydrogenase | 100.00% | 30s879031g005 | | | | 43449 | 31000 | | 6.07% | | 2 | | | ns | | | 2.22 | | ns | |
| 137 | Malate dehydrogenase | 100.00% | 30s892681g001 | | | | 48314 | 68000 | | 6.11% | | 1 | | | 1.57 | | | 6.61 | | 3.52 | |
| 202**^nc^** | Transketolase 1 | 99.00% | 30s656601g005 | | | | 84589 | 95000 | | 1.42% | | 1 | | | 1.86 | | | -8.33 | | -8.45 | |
| 274 | Malate dehydrogenase | 100.00% | 30s892681g001 | | | | 48314 | 65000 | | 7.69% | | 2 | | | ns | | | ns | | 3.30 | |
| 283**^cl^** | Oxygen-evolving enhancer protein chloroplast | 99.00% | 30s991541g001 | | | | 33520 | 23000 | | 4.73% | | 1 | | | ns | | | 2.23 | | -3.29 | |
| 286**^nc^** | Dihydrolipoyllysine-residue succinyltransferase component of 2-oxoglutarate dehydrogenase complex | 99.90% | 30s659921g002 | | | | 39923 | 23000 | | 3.81% | | 1 | | | -1.63 | | | -2.44 | | -1.62 | |
| 403**^nc^** | Citrate synthase | 99.50% | 30s1040901g002 | | | | 30711 | 55000 | | 6.64% | | 1 | | | ns | | | 2.52 | | 3.23 | |
| 412 | Malate dehydrogenase | 100.00% | 30s892681g001 | | | | 48314 | 72000 | | 7.92% | | 3 | | | ns | | | 4.43 | | 5.74 | |
| 421 | Malate dehydrogenase | 100.00% | 30s892681g001 | | | | 48314 | 49000 | | 6.11% | | 1 | | | -1.64 | | | -7.02 | | -4.54 | |
| 428 | Malate dehydrogenase | 100.00% | 30s892681g001 | | | | 48314 | 67000 | | 7.92% | | 2 | | | ns | | | 7.49 | | 5.85 | |
| 443**^nc^** | Dihydrolipoyllysine-residue succinyltransferase component of 2-oxoglutarate dehydrogenase complex | 100.00% | 30s683601g004 | | | | 39697 | 43000 | | 7.14% | | 2 | | | ns | | | -2.25 | | -4.48 | |
| 461 | Malate dehydrogenase | 100.00% | 30s903851g004, 30s946431g012 | | | | 26083 | 30000 | | 4.39% | | 2 | | | ns | | | ns | | -2.09 | |
| 477 | UMP6 mitochondrial precursor | 100.00% | 30s1009951g003 | | | | 16907 | 15000 | | 13.60% | | 2 | | | ns | | | -2.16 | | -2.70 | |
| 482 | Malate dehydrogenase | 100.00% | 30s892681g001 | | | | 48314 | 66000 | | 10.20% | | 4 | | | -2.02 | | | 4.57 | | 5.56 | |
| 503 | Triosephosphate isomerase | 99.20% | 30s678721g005, 30s889891g002 | | | | 27117 | 19000 | | 5.51% | | 1 | | | 2.27 | | | 2.55 | | 2.52 | |
| 506 | Pyruvate dehydrogenase E1 component β-subunit | 100.00% | 30s855801g002 | | | 22740 | | 35000 | | 13.00% | | 2 | | ns | | | -1.60 | | | -1.70 | |
| 523 | Succinyl-CoA ligase β-subunit | 99.90% | 30s732221g008 | | | | 45017 | 43000 | | 7.58% | | 1 | | | ns | | | 1.84 | | 2.64 | |
| 531 | Bisphosphoglycerate-independent phosphoglycerate mutase | 100.00% | 30s661041g002, 30s693071g003 | | | | 43543 | 30000 | | 5.60% | | 1 | | | ns | | | ns | | 1.64 | |
| 555**^cl^** | Oxygen evolving enhancer protein chloroplast | 100.00% | 30s919171g003 | | | | 35305 | 19000 | | 14.70% | | 3 | | | ns | | | -1.68 | | -1.56 | |
| 566 | Fructose-bisphosphate aldolase | 100.00% | 30s1148281g010 | | | | 40222 | 16000 | | 12.10% | | 4 | | | ns | | | -2.03 | | -2.99 | |
| 577 | Transaldolase-like protein | 100.00% | 30s1065691g004 | | | | 43557 | 55000 | | 4.88% | | 2 | | | -1.79 | | | -7.48 | | -4.49 | |
| **03** | **Cell growth/division** |  |  | | | |  |  | |  | |  | | |  | | |  | |  | |
| 45 | Condensin complex subunit 1 | 99.30% | 30s702551g004 | | | | 127265 | 51000 | | 1.47% | | 1 | | | ns | | | 2.53 | | 1.95 | |
| 252 | Enhancer of polycomb-like protein | 99.70% | 30s827291g002 | | | | 51710 | 18000 | | 5.79% | | 1 | | | 4.51 | | | 4.99 | | 2.11 | |
| 366 | Growth regulator | 98.10% | 30s797691g002 | | | | 19927 | 62000 | | 6.29% | | 1 | | | ns | | | ns | | -3.21 | |
| 387 | KU P80 DNA | 99.50% | 30s862811g001 | | | | 75649 | 20000 | | 1.48% | | 1 | | | 2.75 | | | ns | | ns | |
| 415 | 14-3-3-like protein | 99.70% | 30s819041g002 | | | | 17032 | 19000 | | 7.95% | | 1 | | | 2.71 | | | 2.55 | | 1.72 | |
| **04** | **Transcription** |  |  | | | |  |  | |  | |  | | |  | | |  | |  | |
| 302 | Gata transcription factor 25 | 99.70% | 30s1133641g001 | | | | 80728 | 25000 | | 1.37% | | 1 | | | ns | | | 2.14 | | -1.96 | |
| 302 | Inducer of cbf expression 1 DNA binding transcription activator transcription factor | 99.70% | 30s953031g001 | | | | 38914 | 25000 | | 2.51% | | 1 | | | ns | | | 2.14 | | -1.96 | |
| 416 | Transcription factor IIA small subunit | 99.70% | 30s656261g002, 30s760301g006 | | | | 12093 | 27000 | | 10.40% | | 1 | | | 2.52 | | | 3.51 | | 2.46 | |
| 605 | Glycine-rich RNA-binding protein 7 | 99.60% | 30s690791g001 | | | | 21057 | 17000 | | 4.27% | | 1 | | | ns | | | -1.89 | | 1.03 | |
| 611 | Glycine-rich RNA-binding protein 7 | 100.00% | 30s701321g001 | | | | 10258 | 29000 | | 13.50% | | 1 | | | ns | | | ns | | -2.35 | |
| **05** | **Protein synthesis** |  |  | | | |  |  | |  | |  | | |  | | |  | |  | |
| 45 | Translation initiation factor (eif-4a) | 99.90% | 30s724051g001, 30s997411g005 | | | | 47064 | 51000 | | 2.42% | | 1 | | | ns | | | 2.53 | | 1.95 | |
| 53 | Elongation factor 1 | 99.20% | 30s667161g002 | | | | 16163 | 21000 | | 11.90% | | 1 | | | -1.84 | | | -1.79 | | 1.55 | |
| 115 | Elongation factor 1 | 99.60% | 30s1011781g008 | | | | 24433 | 23000 | | 8.04% | | 1 | | | ns | | | -1.71 | | -1.94 | |
| 249 | Gag-pol polyprotein | 99.70% | 30s811431g002 | | | | 49976 | 24000 | | 2.04% | | 1 | | | ns | | | 1.65 | | 1.72 | |
| 274 | Aspartyl-tRNA synthetase | 100.00% | 30s6550950g017 | | | | 66976 | 65000 | | 19.30% | | 7 | | | ns | | | ns | | 3.30 | |
| 287**^nc^** | 30s ribosomal protein s1 | 97.70% | 30s783011g009 | | | | 45242 | 35000 | | 2.15% | | 1 | | | 1.58 | | | 8.73 | | 11.49 | |
| 317 | Translation initiation factor (eif-4a) | 100.00% | 30s997411g005 | | | | 47064 | 45000 | | 21.50% | | 7 | | | ns | | | ns | | -1.56 | |
| 381**^nc^** | 30s ribosomal protein s1 | 99.60% | 30s783011g009 | | | | 45242 | 37000 | | 2.15% | | 1 | | | -1.63 | | | -2.65 | | -2.20 | |
| 416 | Peptide chain release factor, putative | 99.70% | 30s682511g003 | | | | 9163 | 27000 | | 13.30% | | 1 | | | 2.52 | | | 3.51 | | 2.46 | |
| 431 | Elongation factor Tu | 100.00% | 30s812681g001 | | | | 49884 | 39000 | | 24.80% | | 8 | | | ns | | | 4.54 | | 5.18 | |
| 433 | Elongation factor Tu | 100.00% | 30s812681g001 | | | | 49884 | 34000 | | 5.45% | | 1 | | | ns | | | 2.25 | | 2.48 | |
| 504 | 60s ribosomal protein l23a | 99.70% | 30s1035671g001 | | | | 9453 | 88000 | | 11.00% | | 1 | | | 1.62 | | | ns | | -3.63 | |
| 525 | Translation initiation factor (eif-4a) | 100.00% | 30s724051g001, 30s997411g005 | | | | 47064 | 39000 | | 9.93% | | 4 | | | ns | | | 3.98 | | 5.12 | |
| 604 | Translation initiation factor (eif-4a) | 100.00% | 30s724051g001, 30s997411g005 | | | | 47064 | 27000 | | 4.36% | | 2 | | | -1.55 | | | -2.57 | | -1.81 | |
| **06** | **Protein destination and storage** |  |  | | | |  |  | |  | |  | | |  | | |  | |  | |
| 3 | Cysteine protease | 98.80% | 30s790241g001 | | | | 36042 | 22000 | | 4.98% | | 1 | | | ns | | | -2.48 | | -1.80 | |
| 32 | Luminal binding protein | 100.00% | 30s685511g001 | | | | 56109 | 82000 | | 7.24% | | 2 | | | ns | | | ns | | -2.52 | |
| 91 | Chaperonin 21 precursor | 100.00% | 30s720451g001 | | | | 23283 | 19000 | | 9.05% | | 2 | | | 2.09 | | | -1.58 | | -2.61 | |
| 92 | Mitochondrial processing peptidase | 100.00% | 30s927641g002 | | | | 59451 | 55000 | | 6.08% | | 3 | | | ns | | | 1.92 | | 1.88 | |
| 129**^nc^** | Proline iminopeptidase | 100.00% | 30s775611g009 | | | | 40654 | 31000 | | 4.57% | | 2 | | | ns | | | 2.22 | | ns | |
| 190 | Legumain-like protease | 100.00% | 30s799651g001 | | | | 36142 | 22000 | | 2.77% | | 1 | | | 1.52 | | | ns | | -2.77 | |
| 210**^nc^** | Proteasome subunit β type 7-A | 100.00% | 30s1126271g001 | | | | 29520 | 19000 | | 4.40% | | 1 | | | -2.20 | | | -4.21 | | -4.79 | |
| 239 | Luminal binding protein | 100.00% | 30s685511g001 | | | | 56109 | 70000 | | 18.00% | | 8 | | | ns | | | ns | | -1.62 | |
| 288 | Cysteine protease | 99.70% | 30s790241g001 | | | | 36042 | 32000 | | 4.98% | | 1 | | | ns | | | 2.43 | | 2.24 | |
| 292 | Chaperonin 21 precursor | 100.00% | 30s654821g003 | | | | 26999 | 20000 | | 12.80% | | 2 | | | ns | | | -2.11 | | -2.49 | |
| 300 | Nuclear transport | 99.20% | 30s1197741g004 | | | | 16883 | 11000 | | 6.80% | | 1 | | | -2.91 | | | -3.50 | | -1.66 | |
| 302 | Aspartic proteinase nepenthesin-1 precursor | 99.70% | 30s708411g001 | | | | 25085 | 25000 | | 3.85% | | 1 | | | ns | | | 2.14 | | -1.96 | |
| 303 | Subtilisin-like serine proteinase | 100.00% | 30s808251g002 | | | | 48672 | 82000 | | 5.76% | | 2 | | | ns | | | -2.50 | | -1.57 | |
| 331 | TCP 1 CNP60 chaperonin family protein | 100.00% | 30s758931g001 | | | | 54112 | 65000 | | 4.40% | | 2 | | | ns | | | ns | | -1.89 | |
| 336**^nc^** | 26s proteasome AAA-ATPase subunit RPT5a | 100.00% | 30s839411g002 | | | | 47408 | 45000 | | 25.10% | | 10 | | | ns | | | -1.80 | | -2.28 | |
| 370**^nc^** | GDP dissociation inhibitor | 100.00% | 30s1179061g005 | | | | 49697 | 47000 | | 4.50% | | 2 | | | ns | | | 1.74 | | 2.60 | |
| 370**^nc^** | GDP dissociation inhibitor | 100.00% | 30s1179061g005 | | | | 49697 | 47000 | | 4.50% | | 2 | | | ns | | | 1.74 | | 2.60 | |
| 385 | TCP 1 CNP60 chaperonin family protein | 100.00% | 30s758931g001 | | | | 54112 | 61000 | | 6.40% | | 3 | | | ns | | | ns | | -1.91 | |
| 419 | Mitochondrial processing peptidase | 100.00% | 30s927641g002 | | | | 59451 | 55000 | | 4.60% | | 1 | | | ns | | | 2.78 | | ns | |
| 476**^nc^** | 26s proteasome AAA-ATPase subunit RPT5a | 100.00% | 30s839411g002 | | | | 47408 | 45000 | | 9.46% | | 4 | | | ns | | | ns | | -2.83 | |
| 531 | Chaperonin containing t-complex protein epsilon | 100.00% | 30s1179991g005 | | | | 59114 | 30000 | | 7.32% | | 4 | | | ns | | | ns | | 1.64 | |
| 536 | Protein disulfide isomerase | 100.00% | 30s918681g002 | | | | 63245 | 78000 | | 5.74% | | 3 | | | ns | | | -2.13 | | -1.52 | |
| 545 | Protein disulfide isomerase | 100.00% | 30s918681g002 | | | | 63245 | 72000 | | 4.17% | | 2 | | | 1.67 | | | -6.66 | | -2.29 | |
| 555 | Multicatalytic endopeptidase proteasome β-subunit | 100.00% | 30s1065691g004 | | | | 25157 | 19000 | | 34.30% | | 7 | | | ns | | | -1.68 | | -1.56 | |
| 561 | Multicatalytic endopeptidase proteasome β-subunit | 100.00% | 30s1065691g004 | | | | 25157 | 26000 | | 19.30% | | 4 | | | ns | | | ns | | -2.03 | |
| 609 | Cysteine protease | 99.60% | 30s790241g001 | | | | 36042 | 31000 | | 4.98% | | 1 | | | ns | | | -1.86 | | -1.99 | |
| 611 | Cysteine protease | 99.40% | 30s790241g001 | | | | 36042 | 29000 | | 4.98% | | 1 | | | ns | | | ns | | -2.35 | |
| 614**^nc^** | Proteasome subunit β type 2 | 95.20% | 30s943301g033 | | | | 18869 | 38000 | | 5.33% | | 1 | | | ns | | | -8.15 | | -1.79 | |
| 632**^nc^** | Proteasome subunit α- type 2 | 99.60% | 30s1154301g003 | | | | 25656 | 19000 | | 4.68% | | 1 | | | ns | | | 1.68 | | 9.34 | |
| **07** | **Transporters** |  |  | | | |  |  | |  | |  | | |  | | |  | |  | |
| 99 | Vacuolar ATP synthase subunit v- proton pump 57 kda | 100.00% | 30s735571g002, 30s837971g002 | | | | 58290 | 58000 | | 3.08% | | 2 | | | ns | | | 2.13 | | ns | |
| 312 | ATP synthase β chain | 100.00% | 30s884401g004 | | | | 55268 | 54000 | | 5.10% | | 2 | | | ns | | | -2.28 | | -1.65 | |
| 403 | CMP-sialic acid | 99.90% | 30s808171g003 | | | | 23646 | 55000 | | 9.52% | | 1 | | | ns | | | 2.52 | | 3.23 | |
| 410 | Vacuolar H+-ATPase catalytic subunit | 100.00% | 30s1095631g001 | | | | 68335 | 70000 | | 4.99% | | 4 | | | ns | | | 2.16 | | 1.84 | |
| 419 | ATP synthase β chain | 100.00% | 30s884401g004 | | | | 55268 | 55000 | | 7.25% | | 3 | | | ns | | | 2.78 | | ns | |
| 468 | Mitochondrial deoxynucleotide carrier, putative | 99.70% | 30s656531g003 | | 36808 | | | 16000 | | 2.69% | | 1 | | ns | | | -2.26 | | | -2.47 | |
| 507 | ATP synthase β chain | 99.50% | 30s884401g004 | | | | 55268 | 43000 | | 2.75% | | 1 | | | ns | | | 3.96 | | 9.99 | |
| **08** | **Intracellular traffic** |  |  | | | |  |  | |  | |  | | |  | | |  | |  | |
| 417 | Protein binding structural molecule | 99.90% | 30s784571g001 | | | 33386 | | 50000 | | 6.89% | | 1 | | | ns | | | -1.93 | | -2.51 | |
| **09** | **Cell structure** |  |  | | | |  |  | |  | |  | | |  | | |  | |  | |
| 3 | Fibrillin-like protein | 98.80% | 30s724791g001, 30s837971g003 | | | | 39091 | 22000 | | 4.19% | | 1 | | | ns | | | -2.48 | | -1.80 | |
| 14**^nc^** | Tubulin β-chain | 100.00% | 30s770201g001, 30s837411g003, 30s965611g001 | | | | 50234 | 57000 | | 13.90% | | 1 | | | ns | | | -2.38 | | -2.52 | |
| 19**^nc^** | Plastid-lipid associated protein 3 | 98.30% | 30s761251g002 | | | | 20316 | 37000 | | 5.35% | | 1 | | | ns | | | -1.61 | | -2.37 | |
| 53 | Fibrillin-like protein | 99.20% | 30s724791g001, 30s837971g003 | | | | 39091 | 21000 | | 4.19% | | 1 | | | -1.84 | | | -1.79 | | 1.55 | |
| 56**^nc^** | Tubulin β-2/β-3 chain | 99.40% | 30s837411g003 | | | | 50234 | 57000 | | 8.04% | | 1 | | | ns | | | -2.75 | | -3.43 | |
| 56**^nc^** | Tubulin β-chain | 100.00% | 30s708731g001 | | | | 50095 | 57000 | | 11.50% | | 1 | | | ns | | | -2.75 | | -3.43 | |
| 129**^nc^** | Actin | 100.00% | 30s794481g001, 30s929831g004 | | | | 41692 | 31000 | | 13.50% | | 2 | | | ns | | | 2.22 | | ns | |
| 189 | α- tubulin | 99.40% | 30s1145261g002, 30s1190081g004, 30s6550926g030, 30s831221g002, 30s870011g003 | | | | 51860 | 51000 | | 2.22% | | 1 | | | ns | | | -1.83 | | -6.85 | |
| 197**^nc^** | Tubulin β-chain | 100.00% | 30s708731g001 | | | | 50095 | 61000 | | 10.60% | | 1 | | | ns | | | ns | | -7.28 | |
| 317**^nc^** | Actin | 100.00% | 30s1070141g012, 30s717671g011, 30s844721g006, 30s951221g005 | | | | 41610 | 45000 | | 22.30% | | 3 | | | ns | | | ns | | -1.56 | |
| 356**^nc^** | Tubulin β-2 β-3 chain | 100.00% | 30s837411g003, 30s965611g001 | | | | 50234 | 34000 | | 9.60% | | 1 | | | ns | | | ns | | 2.39 | |
| 356**^nc^** | Tubulin β-chain | 100.00% | 30s1088541g002, 30s697331g005, 30s708731g001 | | | | 50095 | 34000 | | 9.60% | | 1 | | | ns | | | ns | | 2.39 | |
| 359**^nc^** | α- tubulin 1 | 100.00% | 30s831221g002, 30s870011g003 | | | | 36106 | 51000 | | 9.97% | | 4 | | | ns | | | -2.42 | | -5.47 | |
| 400 | Actin depolymerizing factor | 99.40% | 30s1070141g030, 30s65509449g002, 30s845821g004 | | | | 15349 | 13000 | | 6.82% | | 1 | | | 3.67 | | | 1.63 | | ns | |
| 417**^nc^** | α- tubulin 1 | 100.00% | 30s831221g002 | | | | 47651 | 50000 | | 11.60% | | 1 | | | ns | | | -1.93 | | -2.51 | |
| 417**^nc^** | α- tubulin 1 | 100.00% | 30s6550926g030 | | | | 51860 | 50000 | | 10.60% | | 1 | | | ns | | | -1.93 | | -2.51 | |
| 422**^nc^** | Tubulin β-chain | 99.90% | 30s708731g001 | | | | 50095 | 55000 | | 5.62% | | 1 | | | ns | | | -2.17 | | -1.99 | |
| 487**^nc^** | Actin | 100.00% | 30s1070141g012, 30s717671g011, 30s844721g006, 30s951221g005 | | | | 41610 | 39000 | | 29.20% | | 1 | | | ns | | | 2.13 | | 1.75 | |
| 608 | Translationally controlled tumor protein | 99.60% | 30s702241g005, 30s703891g002, 30s764761g001, 30s828021g001 | | | | 19074 | 31000 | | 5.36% | | 1 | | | ns | | | 1.92 | | 1.87 | |
| **10** | **Signal transduction** | | | | | | | | | | | | | | | | | | | | |
| 34 | Germin-like protein | 98.10% | | 30s1014231g001 | | | 13337 | | 18000 | | 17.60% | | 1 | | | 2.71 | | | 5.01 | | 4.50 |
| 64 | Adenosine kinase | 100.00% | | 30s970341g005 | | | 37395 | | 31000 | | 11.10% | | 3 | | | ns | | | -1.67 | | -1.71 |
| 91 | Uridylate kinase | 100.00% | | 30s1123951g008 | | | 23322 | | 19000 | | 21.00% | | 4 | | | 2.09 | | | -1.58 | | -2.61 |
| 96 | Nucleoside diphosphate kinase | 100.00% | | 30s835621g002 | | | 12145 | | 16000 | | 28.80% | | 3 | | | 1.74 | | | -1.50 | | -1.62 |
| 114 | Nucleoside diphosphate kinase | 96.00% | | 30s6550969g003 | | | 16496 | | 12000 | | 16.90% | | 0 | | | ns | | | 1.95 | | 2.43 |
| 203 | Nucleoside diphosphate kinase | 99.80% | | 30s835621g002 | | | 24174 | | 23000 | | 36.00% | | 1 | | | 1.66 | | | 2.11 | | 1.99 |
| 272 | Uridylate kinase | 100.00% | | 30s1123951g008 | | | 23322 | | 20000 | | 10.50% | | 3 | | | ns | | | -5.09 | | -4.71 |
| 294 | Nucleoside diphosphate kinase | 100.00% | | 30s6550969g003 | | | 16496 | | 12000 | | 32.40% | | 1 | | | ns | | | 1.62 | | 1.88 |
| 314 | IN2-1 protein | 99.80% | | 30s696261g003 | | | 37485 | | 19000 | | 2.73% | | 1 | | | -1.83 | | | ns | | 6.10 |
| 431 | Phosphoglycerate kinase | 100.00% | | 30s1078131g002 | | | 15880 | | 39000 | | 9.40% | | 2 | | | ns | | | 4.54 | | 5.18 |
| 431 | Phosphoglycerate kinase | 100.00% | | 30s724451g002 | | | 42301 | | 39000 | | 5.00% | | 2 | | | ns | | | 4.54 | | 5.18 |
| 431**^nc^** | Mitochondrial pyruvate dehydrogenase kinase isoform 1 | 100.00% | | 30s1002501g002 | | | 38670 | | 39000 | | 7.25% | | 2 | | | ns | | | 4.54 | | 5.18 |
| 555 | GTP binding protein | 99.90% | | 30s952801g001 | | | 16592 | | 19000 | | 8.67% | | 1 | | | ns | | | -1.68 | | -1.56 |
| 587**^nc^** | Zinc finger | 100.00% | | 30s796661g001 | | | 41956 | | 35000 | | 5.15% | | 2 | | | -1.85 | | | -8.66 | | -6.63 |
| **11** | **Disease/defence** |  | |  | | |  | |  | |  | |  | | |  | | |  | |  |
| 13**^nc^** | Leucine rich repeat-containing | 100.00% | | 30s857351g006 | | | 49345 | | 64000 | | 9.64% | | 3 | | | ns | | | ns | | -1.95 |
| 17 | ABA-hypersensitive germination 2 nucleic acid binding ribonuclease | 99.10% | | 30s1120391g003 | | | 79218 | | 75000 | | 1.44% | | 1 | | | -2.68 | | | 5.86 | | 2.68 |
| 26 | Heat shock protein | 100.00% | | 30s897151g002 | | | 72180 | | 72000 | | 10.10% | | 3 | | | ns | | | ns | | -3.10 |
| 32**^nc^** | Heat shock protein 70 | 100.00% | | 30s941391g004 | | | 71057 | | 82000 | | 10.90% | | 3 | | | ns | | | ns | | -2.52 |
| 32 | Heat shock protein | 99.90% | | 30s1034811g002 | | | 131443 | | 82000 | | 4.99% | | 1 | | | ns | | | ns | | -2.52 |
| 40 | HSP associated protein like | 99.40% | | 30s828861g001 | | | 28609 | | 53000 | | 3.50% | | 2 | | | -1.86 | | | -3.34 | | -5.06 |
| 70**^cl^** | Aldo/keto reductase | 98.80% | | 30s1183861g001 | | | 38732 | | 44000 | | 4.82% | | 1 | | | -1.97 | | | -2.16 | | -1.52 |
| 81 | Glutathione transferase | 100.00% | | 30s806101g003 | | | 37154 | | 30000 | | 8.00% | | 2 | | | ns | | | ns | | 1.67 |
| 210 | Glutathione s-transferase GSTF2 | 100.00% | | 30s987821g001 | | | 13597 | | 19000 | | 19.80% | | 2 | | | -2.20 | | | -4.21 | | -4.79 |
| 239**^nc^** | Heat shock protein 70 | 100.00% | | 30s795061g002 | | | 71222 | | 70000 | | 19.40% | | 1 | | | ns | | | ns | | -1.62 |
| 239**^nc^** | Heat shock protein 70 | 100.00% | | 30s941391g005 | | | 61596 | | 70000 | | 18.10% | | 3 | | | ns | | | ns | | -1.62 |
| 239**^nc^** | Heat shock protein 70 | 100.00% | | 30s941391g004 | | | 71057 | | 70000 | | 15.10% | | 1 | | | ns | | | ns | | -1.62 |
| 239**^nc^** | Heat shock cognate 70 kda expressed | 99.80% | | 30s1034811g003 | | | 13813 | | 70000 | | 17.60% | | 1 | | | ns | | | ns | | -1.62 |
| 246 | ABA-hypersensitive germination 2 nucleic acid binding ribonuclease | 98.70% | | 30s1120391g003 | | | 79218 | | 22000 | | 1.44% | | 1 | | | ns | | | -1.50 | | -2.15 |
| 274**^nc^** | Leucine-rich repeat transmembrane protein | 100.00% | | 30s6550960g006 | | | 42291 | | 65000 | | 23.00% | | 4 | | | ns | | | ns | | 3.30 |
| 274**^nc^** | Leucine-rich repeat transmembrane protein | 100.00% | | 30s6550960g006 | | | 42291 | | 65000 | | 12.70% | | 2 | | | ns | | | ns | | 3.30 |
| 274**^cl^** | Aldo/keto reductase | 100.00% | | 30s1183861g001 | | | 38732 | | 65000 | | 10.50% | | 3 | | | ns | | | ns | | 3.30 |
| 303 | Heat shock protein 82 | 100.00% | | 30s947641g003 | | | 80204 | | 82000 | | 10.70% | | 2 | | | ns | | | -2.50 | | -1.57 |
| 303 | Heat shock protein | 100.00% | | 30s1195181g002 | | | 80554 | | 82000 | | 10.10% | | 5 | | | ns | | | -2.50 | | -1.57 |
| 303 | Heat shock protein 82 | 100.00% | | 30s722381g008 | | | 89708 | | 82000 | | 12.40% | | 3 | | | ns | | | -2.50 | | -1.57 |
| 326 | Heat shock protein | 99.90% | | 30s705951g009 | | | 60083 | | 78000 | | 6.23% | | 1 | | | ns | | | -1.68 | | -3.69 |
| 326 | Heat shock protein | 100.00% | | 30s897151g002 | | | 72180 | | 78000 | | 7.03% | | 4 | | | ns | | | -1.68 | | -3.69 |
| 344 | Heat shock protein 82 | 99.60% | | 30s1082281g001, 30s722381g008, 30s947641g003 | | | 89708 | | 80000 | | 3.17% | | 1 | | | ns | | | -2.73 | | -1.57 |
| 352 | Heat shock protein 82 | 99.70% | | 30s1082281g001, 30s722381g008, 30s947641g003 | | | 89708 | | 54000 | | 3.17% | | 1 | | | ns | | | ns | | -2.00 |
| 357 | Glyoxalase i homolog (ATGLX1) | 99.70% | | 30s1166451g001 | | | 16766 | | 24000 | | 8.97% | | 1 | | | ns | | | 1.75 | | 1.91 |
| 362**^nc^** | Heat shock protein 70 | 100.00% | | 30s941391g005 | | | 61596 | | 70000 | | 5.90% | | 2 | | | ns | | | 1.83 | | -3.04 |
| 362**^nc^** | Heat shock protein 70 | 100.00% | | 30s795061g002 | | | 71222 | | 70000 | | 6.02% | | 3 | | | ns | | | 1.83 | | -3.04 |
| 378**^cl^** | Aldo/keto reductase | 99.80% | | 30s1183861g001 | | | 38732 | | 65000 | | 2.27% | | 1 | | | ns | | | ns | | 3.09 |
| 380**^nc^** | E3 ubiquitin ligase | 99.60% | | 30s1006041g001 | | | 121028 | | 18000 | | 0.91% | | 1 | | | 2.31 | | | 2.33 | | 1.68 |
| 401 | Aldose reductase | 100.00% | | 30s1006721g002 | | | 34679 | | 19000 | | 14.10% | | 5 | | | 2.13 | | | ns | | 1.78 |
| 403 | Quinone oxidoreductase-like protein | 100.00% | | 30s1122781g002 | | | 33365 | | 55000 | | 7.54% | | 2 | | | ns | | | 2.52 | | 3.23 |
| 403 | Catalase | 100.00% | | 30s1080231g005 | | | 55425 | | 55000 | | 10.70% | | 5 | | | ns | | | 2.52 | | 3.23 |
| 404 | Catalase | 100.00% | | 30s1080231g005 | | | 55425 | | 55000 | | 4.62% | | 2 | | | ns | | | 3.79 | | 3.82 |
| 461**^cl^** | Aldo/keto reductase | 100.00% | | 30s1183861g001 | | | 38732 | | 30000 | | 22.10% | | 7 | | | ns | | | ns | | -2.09 |
| 503 | Glutathione s-transferase GSTF2 | 100.00% | | 30s987821g001 | | | 13597 | | 19000 | | 14.90% | | 2 | | | 2.27 | | | 2.55 | | 2.52 |
| 511**^nc^** | Heat shock protein 70 | 99.80% | | 30s941391g005 | | | 61596 | | 70000 | | 3.76% | | 1 | | | ns | | | 2.18 | | -2.14 |
| 511 | Heat shock protein | 100.00% | | 30s897151g002 | | | 72180 | | 70000 | | 2.78% | | 2 | | | ns | | | 2.18 | | -2.14 |
| 522**^cl^** | Aldo/keto reductase | 100.00% | | 30s1183861g001 | | | 38732 | | 37000 | | 37.40% | | 17 | | | ns | | | -2.27 | | -3.20 |
| 523**^cl^** | Aldo/keto reductase | 100.00% | | 30s1183861g001 | | | 38732 | | 43000 | | 11.90% | | 3 | | | ns | | | 1.84 | | 2.64 |
| 525**^cl^** | Aldo/keto reductase | 100.00% | | 30s1183861g001 | | | 38732 | | 39000 | | 9.07% | | 2 | | | ns | | | 3.98 | | 5.12 |
| 529**^cl^** | Aldo/keto reductase | 100.00% | | 30s1183861g001 | | | 38732 | | 64000 | | 9.07% | | 2 | | | ns | | | -1.71 | | -1.96 |
| 566**^cl^** | Aldo/keto reductase | 99.50% | | 30s1183861g001 | | | 38732 | | 16000 | | 4.82% | | 1 | | | ns | | | -2.03 | | -2.99 |
| 567**^cl^** | Aldo/keto reductase | 99.60% | | 30s1109381g003 | | | 31107 | | 20000 | | 5.09% | | 1 | | | 2.04 | | | ns | | -2.24 |
| 583**^nc^** | E3 ubiquitin ligase | 99.70% | | 30s1065691g004 | | | 121028 | | 46000 | | 0.91% | | 1 | | | ns | | | -5.11 | | -6.36 |
| 660 | USP family protein | 99.60% | | 30s728721g001, 30s768591g001, 30s938201g006 | | | 21181 | | 25000 | | 5.61% | | 1 | | | ns | | | ns | | -2.64 |
| **12** | **Unclear classification** |  | |  | | |  | |  | |  | |  | | |  | | |  | |  |
| 91 | Riboflavin α- subunit | 99.00% | | 30s703111g002 | | | 28760 | | 19000 | | 5.22% | | 2 | | | 2.09 | | | -1.58 | | -2.61 |
| 129 | Epoxide expressed | 100.00% | | 30s749161g001 | | | 43400 | | 31000 | | 10.90% | | 4 | | | ns | | | 2.22 | | ns |
| 199 | Nucleic acid binding | 99.80% | | 30s1050381g022 | | | 36142 | | 22000 | | 6.98% | | 1 | | | ns | | | -2.95 | | -2.56 |
| 477 | Nucleic acid binding | 100.00% | | 30s1050381g022 | | | 14374 | | 15000 | | 10.90% | | 2 | | | ns | | | -2.16 | | -2.70 |
| 507 | JHL06P13.3 like protein | 99.80% | | 30s1042771g010 | | | 50733 | | 43000 | | 2.22% | | 1 | | | ns | | | 3.96 | | 9.99 |
| 508 | JHL06P13.3 like protein | 100.00% | | 30s1042771g010 | | | 50733 | | 55000 | | 4.44% | | 2 | | | ns | | | 1.73 | | ns |
| 561 | Mitochondrial glycoprotein | 99.20% | | 30s796661g001 | | | 33383 | | 26000 | | 4.05% | | 1 | | | ns | | | ns | | -2.03 |
| **14** | **Unclassified** | | | | | | | | | | | | | | | | | | | | |
| 13 | Partial gene, unknown protein | 100.00% | | 30s1131351g004 | | | 51113 | | 64000 | | 22.30% | | 6 | | | ns | | | ns | | -1.95 |
| 62 | Partial gene, unknown protein | 96.00% | | 30s685821g001 | | | 14268 | | 15000 | | 6.35% | | 1 | | | 2.67 | | | 4.90 | | ns |
| 80 | Partial gene, unknown protein | 97.30% | | 30s1164091g001 | | | 48663 | | 51000 | | 1.86% | | 1 | | | ns | | | -4.53 | | -3.84 |
| 89 | Similar to predicted protein | 100.00% | | 30s874781g004 | | | 64668 | | 62000 | | 5.26% | | 3 | | | ns | | | ns | | -2.24 |
| 93 | Partial gene, unknown protein | 96.60% | | 30s65509675g001 | | | 96099 | | 20000 | | 2.12% | | 1 | | | 5.15 | | | 2.98 | | 1.99 |
| 109 | Similar to predicted protein | 99.70% | | 30s1025991g001 | | | 27229 | | 21000 | | 6.20% | | 1 | | | -2.17 | | | ns | | ns |
| 120 | Partial gene, unknown protein | 98.50% | | 30s65509404g001 | | | 55471 | | 30000 | | 2.39% | | 1 | | | -2.15 | | | -2.56 | | 1.77 |
| 122 | Partial gene, unknown protein | 95.70% | | 30s654701g002 | | | 34756 | | 14000 | | 4.40% | | 1 | | | ns | | | -3.12 | | -1.67 |
| 128 | Partial gene, unknown protein | 97.10% | | 30s6550976g004 | | | 18657 | | 15000 | | 5.03% | | 1 | | | -1.79 | | | 11.49 | | ns |
| 138 | Partial gene, unknown protein | 98.20% | | 30s738571g003 | | | 24648 | | 92000 | | 4.19% | | 1 | | | -1.92 | | | -6.31 | | -3.21 |
| 155 | Partial gene, unknown protein | 97.60% | | 30s738571g003 | | | 24648 | | 11000 | | 4.19% | | 1 | | | ns | | | ns | | 2.15 |
| 177 | Similar to predicted protein | 100.00% | | 30s1112311g003 | | | 40944 | | 34000 | | 7.34% | | 2 | | | ns | | | 1.73 | | 2.19 |
| 195 | Partial gene, unknown protein | 97.90% | | 30s1059561g002 | | | 84589 | | 95000 | | 1.04% | | 1 | | | ns | | | -3.83 | | -1.83 |
| 222 | Partial gene, unknown protein | 96.90% | | 30s65509404g001 | | | 55471 | | 22000 | | 2.39% | | 1 | | | 4.04 | | | 2.18 | | 3.08 |
| 267 | Partial gene, unknown protein | 95.50% | | 30s762331g001, 30s963831g001 | | | 22339 | | 20000 | | 4.12% | | 1 | | | -1.71 | | | -2.50 | | ns |
| 279 | Partial gene, unknown protein | 96.70% | | 30s65509404g001 | | | 55471 | | 50000 | | 2.39% | | 1 | | | ns | | | -2.28 | | -4.43 |
| 295 | Partial gene, unknown protein | 95.40% | | 30s1074861g022 | | | 57460 | | 31000 | | 3.65% | | 1 | | | -1.85 | | | -1.73 | | ns |
| 299 | Partial gene, unknown protein | 98.40% | | 30s897561g001 | | | 54787 | | 20000 | | 5.20% | | 1 | | | -1.95 | | | ns | | 5.99 |
| 337 | Partial gene, unknown protein | 99.30% | | 30s871741g001 | | | 35593 | | 14000 | | 3.36% | | 1 | | | 1.80 | | | 2.25 | | 1.88 |
| 338 | Partial gene, unknown protein | 99.20% | | 30s674341g002, 30s760781g005 | | | 18500 | | 22000 | | 6.28% | | 1 | | | ns | | | -1.93 | | -2.25 |
| 351 | Similar to predicted protein | 98.80% | | 30s65509287g003 | | | 48098 | | 65000 | | 1.83% | | 1 | | | ns | | | 2.20 | | 4.34 |
| 366 | Partial gene, unknown protein | 98.10% | | 30s852711g001 | | | 9619 | | 62000 | | 15.70% | | 1 | | | ns | | | ns | | -3.21 |
| 398 | Similar to predicted protein | 99.50% | | 30s763631g009 | | | 108388 | | 10000 | | 1.03% | | 1 | | | 1.83 | | | 2.79 | | 4.34 |
| 447 | Partial gene, unknown protein | 99.70% | | 30s1005041g004 | | | 32483 | | 60000 | | 4.18% | | 1 | | | ns | | | -2.31 | | -7.86 |
| 447 | Partial gene, unknown protein | 99.70% | | 30s1034291g001 | | | 21961 | | 60000 | | 6.67% | | 1 | | | ns | | | -2.31 | | -7.86 |
| 468 | Similar to predicted protein | 99.70% | | 30s656531g003 | | | 36808 | | 16000 | | 2.69% | | 1 | | | ns | | | -2.26 | | -2.47 |
| 468 | Partial gene, unknown protein | 99.70% | | 30s711951g001, 30s721361g001 | | | 42826 | | 16000 | | 3.54% | | 1 | | | ns | | | -2.26 | | -2.47 |
| 477 | Partial gene, unknown protein | 100.00% | | 30s736491g004 | | | 17410 | | 15000 | | 25.90% | | 4 | | | ns | | | -2.16 | | -2.70 |
| 482 | Similar to predicted protein | 100.00% | | 30s920331g001 | | | 61203 | | 66000 | | 7.16% | | 4 | | | -2.02 | | | 4.57 | | 5.56 |
| 504 | Partial gene, unknown protein | 99.70% | | 30s1121471g002 | | | 16498 | | 88000 | | 7.59% | | 1 | | | 1.62 | | | ns | | -3.63 |
| 506 | Similar to predicted protein | 100.00% | | 30s855801g002 | | | 22740 | | 35000 | | 13.00% | | 2 | | | ns | | | -1.60 | | -1.70 |
| 531 | Partial gene, unknown protein | 100.00% | | 30s1178641g002 | | | 18049 | | 30000 | | 32.10% | | 7 | | | ns | | | ns | | 1.64 |
| 551 | Similar to predicted protein | 100.00% | | 30s705371g004 | | | 60615 | | 55000 | | 1.80% | | 1 | | | ns | | | 1.94 | | 1.53 |
| 554 | Similar to predicted protein | 100.00% | | 30s917631g001 | | | 53492 | | 1500 | | 4.30% | | 2 | | | 4.35 | | | ns | | ns |
| 567 | Similar to predicted protein | 99.90% | | 30s1074611g001 | | | 20632 | | 20000 | | 16.70% | | 1 | | | 2.04 | | | ns | | -2.24 |
| 568 | Similar to predicted protein | 99.90% | | 30s1074611g001 | | | 20632 | | 33000 | | 4.43% | | 1 | | | ns | | | -2.27 | | -23.17 |
| 583 | Partial gene, unknown protein | 99.70% | | 30s95801g001 | | | 29765 | | 46000 | | 4.04% | | 1 | | | ns | | | -5.11 | | -6.36 |
| 600 | Partial gene, unknown protein | 99.70% | | 30s726351g001 | | | 13213 | | 39000 | | 10.50% | | 1 | | | ns | | | 10.44 | | 15.57 |
| 600 | Similar to predicted protein | 99.70% | | 30s955011g001 | | | 16518 | | 39000 | | 7.19% | | 1 | | | ns | | | 10.44 | | 15.57 |
| 609 | Partial gene, unknown protein | 99.50% | | 30s735541g002 | | | 21384 | | 31000 | | 4.98% | | 2 | | | ns | | | -1.86 | | -1.99 |
| 629 | Unknown protein (CYP) | 99.60% | | 30s910881g006 | | | 30631 | | 21000 | | 2.79% | | 1 | | | -2.12 | | | ns | | -1.68 |
| **20** | **Secondary metabolism** |  | |  | | |  | |  | |  | |  | | |  | | |  | |  |
| 67 | Caffeoyl CoA O-methyltransferase 1 | 99.00% | | 30s782571g006 | | | 28919 | | 22000 | | 4.65% | | 1 | | | ns | | | ns | | 1.97 |
| 70**^nc^** | Chalcone synthase | 100.00% | | 30s1024681g001 | | | 35823 | | 44000 | | 6.10% | | 2 | | | -1.97 | | | -2.16 | | -1.52 |
| 63**^nc^** | Isopentenyl diphosphate isomerase | 100.00% | | 30s802901g001 | | | 32219 | | 20000 | | 6.86% | | 2 | | | 1.75 | | | ns | | -2.50 |
| 63**^nc^** | Isopentenyl pyrophosphate isomerase | 100.00% | | 30s762371g001 | | | 17552 | | 20000 | | 13.10% | | 2 | | | 1.75 | | | ns | | -2.50 |
| 69**^cl^** | Anthocyanidin synthase | 100.00% | | 30s1125611g001 | | | 38960 | | 39000 | | 2.61% | | 2 | | | 1.59 | | | 21.25 | | 22.05 |
| 91**^nc^** | Chalcone isomerase | 100.00% | | 30s1070141g002 | | | 24742 | | 19000 | | 4.27% | | 1 | | | 2.09 | | | -1.58 | | -2.61 |
| 98**^nc^** | TPA: isoflavone reductase-like protein 5 | 100.00% | | 30s790251g002 | | | 33763 | | 25000 | | 12.00% | | 4 | | | ns | | | 2.27 | | 2.81 |
| 272**^nc^** | Chalcone isomerase | 100.00% | | 30s1070141g002 | | | 24742 | | 20000 | | 24.80% | | 6 | | | ns | | | -5.09 | | -4.71 |
| 274**^nc^** | Chalcone isomerase | 100.00% | | 30s1070141g002 | | | 24742 | | 65000 | | 14.50% | | 3 | | | ns | | | ns | | 3.30 |
| 286**^cl^** | Anthocyanidin synthase | 100.00% | | 30s1125611g001 | | | 38960 | | 23000 | | 5.51% | | 4 | | | -1.63 | | | -2.44 | | -1.62 |
| 374 | Chalcone-flavonone isomerase | 99.90% | | 30s680691g004 | | | 22634 | | 19000 | | 5.94% | | 1 | | | ns | | | -2.81 | | -1.53 |
| 431**^nc^** | Glutamate 1-semialdehyde aminotransferase | 100.00% | | 30s1039641g001 | | | 50533 | | 39000 | | 5.29% | | 2 | | | ns | | | 4.54 | | 5.18 |
| 603**^nc^** | Glutamate 1-semialdehyde aminotransferase | 99.60% | | 30s1039641g001 | | | 50533 | | 55000 | | 2.54% | | 1 | | | ns | | | 4.07 | | 3.70 |
| 623 | Formamidase-like protein | 99.50% | | 30s702741g005 | | | 48663 | | 34000 | | 2.47% | | 1 | | | ns | | | -1.73 | | -3.02 |
| 635 | Formamidase-like protein | 99.50% | | 30s702741g005 | | | 48663 | | 54000 | | 2.47% | | 1 | | | -1.66 | | | -3.63 | | -4.27 |

NUP-number of unique peptides, FC-fold change, ns-not significant, **^cl^-**protein names that have been detected specific to climacteric ripening process,^95^ **^nc^**- protein names specific to non-climacteric ripening process.^95^
