# Supplementary figures and images for: Dynamic changes in the date palm fruit proteome during development and ripening
Source: Hortic Res. 2014 Aug 6;1:14039–. doi: 10.1038/hortres.2014.39 (PMC4596323; doi:10.1038/hortres.2014.39)

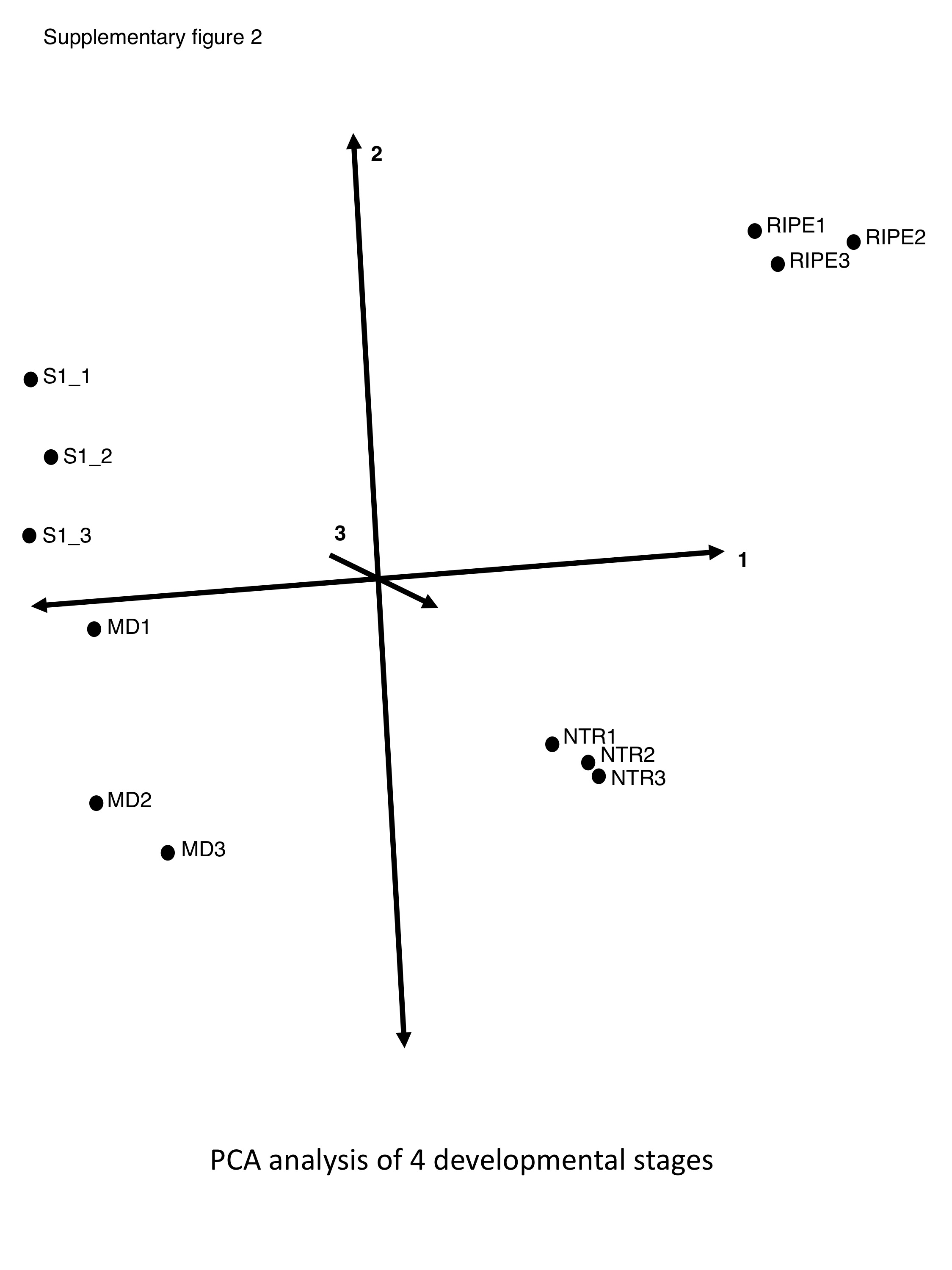

Supplement: Supplementary File 1 [file hortres201439-s2.jpg]
